# Supplementary material for: Characteristics and Components of Self-Management Interventions for Improving Quality of Life in Cancer Survivors: A Systematic Review
Source: Cancers (Basel). 2023 Dec 19;16(1):14. doi: 10.3390/cancers16010014 (PMC10777971; doi:10.3390/cancers16010014)
Supplement: Supplementary file 1 [file cancers-16-00014-s001.zip › cancers-2718461-supplementary/cancers-2718461-supplementary/Supplementary tables.pdf]

Characteristics and components of self-management interventions for improving quality of life in cancer survivors: a systematic review

Ben Rimmer[1,2*], Morven C Brown[1,2], Tumi Sotire[2], Fiona Beyer[2], Iakov Bolnykh[3], Michelle Balla[3], Catherine Richmond[2], Lizzie Dutton[1,2], Sophie Williams[4], Vera Araújo-Soares[1,2,5], Tracy Finch[6], Pamela Gallagher[7], Joanne Lewis[4], Richéal Burns[8,9], Linda Sharp[1,2]

Author affiliations:

1. *Newcastle University Centre for Cancer, Newcastle University, Newcastle upon Tyne, England*
2. *Population Health Sciences Institute, Newcastle University, Newcastle upon Tyne, England*
3. *Faculty of Medical Sciences, Newcastle University, Newcastle upon Tyne, England*
4. *Newcastle upon Tyne Hospitals NHS Foundation Trust, Newcastle upon Tyne, England*
5. *Centre for Preventive Medicine and Digital Health, Department for Prevention, Medical Faculty Mannheim, Heidelberg University, Heidelberg, Germany*
6. *Department of Nursing, Midwifery and Health, Northumbria University, Newcastle upon Tyne, England*
7. *School of Psychology, Dublin City University, Dublin, Ireland*
8. *Faculty of Science, Atlantic Technological University, Sligo, Ireland*
9. *Health and Biomedical Strategic Research Centre, Atlantic Technological University, Ireland*

**Corresponding author: Ben Rimmer, Population Health Sciences Institute, Newcastle University, Ridley Building 1, Newcastle upon Tyne, England, NE1 7RU.*

Email: ben.rimmer@newcastle.ac.uk; Phone: 0044 (0)7704 300 509

Contents

Table S1: Search concepts....................2
Table S2: Database searches....................2
Table S3: Protocols and papers to support intervention development....................13
Table S4: Additional population characteristics of cancer survivors....................15
Table S5: Theory and rationale for the intervention....................19
Table S6: Lorig and Holman self-management tasks....................22
Table S7: Implementation issues....................27
Table S8: Critical appraisal skills programme (CASP) risk of bias appraisal....................34
Table S9: Joanna Briggs Institute (JBI) risk of bias appraisal....................36
Table S10: Quality of life instruments and their scoring....................37
Table S11: Self-efficacy and additional outcomes....................41
Table S12: Economics....................47
Supplementary data....................48

Table S1: Search concepts

	Key concept	Keywords
1.	Cancer	Cancer OR neoplasms OR tumours OR malignancies OR carcinoma
2.	Survivorship	Survivors OR post treatment OR after treatment OR aftercare OR survivorship
3.	Self-management	Self-management OR self-care OR self-efficacy OR self-help OR self-guided OR self-directed OR self-regulated
4.	Intervention	Intervention OR programme OR care package
5.	Evaluation	Feasibility OR acceptability OR efficacy OR effectiveness OR training OR education OR evaluation OR strategies OR pilot OR economics

Table S2: Database searches

Table S2a: MEDLINE

1 cancer.ti,ab,kw. or exp Neoplasms/
2 neoplasm*.ti,ab,kw.
3 (tumor* or tumour*).ti,ab,kw.
4 malignan*.ti,ab,kw.
5 exp CARCINOMA/ or carcinoma*.ti,ab,kw.
6 or/1-5
7 exp SURVIVORS/ or exp CANCER SURVIVORS/ or survivor*.ti,ab,kw.
8 "post treat*".ti,ab,kw.
9 "after treat*".ti,ab,kw.
10 (treat* adj2 complet*).ti,ab,kw.
11 aftercare.ti,ab,kw. or exp AFTERCARE/
12 exp SURVIVORSHIP/
13 or/7-12
14 "self manage*".ti,ab,kw. or exp Self-Management/
15 "self care".ti,ab,kw. or exp Self Care/
16 "self efficacy".ti,ab,kw. or exp Self Efficacy/
17 "self help".ti,ab,kw.
18 "self guid*".ti,ab,kw.
19 "self direct*".ti,ab,kw.
20 "self regulat*".ti,ab,kw.
21 or/14-20
22 intervention*.ti,ab,kw.
23 program*.ti,ab,kw.
24 (care adj3 package*).ti,ab,kw.
25 feasib*.ti,ab,kw.
26 acceptab*.ti,ab,kw.
27 efficacy.ti,ab,kw.
28 effective*.ti,ab,kw.
29 training.ti,ab,kw.
30 educat*.ti,ab,kw.
31 evaluat*.ti,ab,kw.
32 strateg*.ti,ab,kw.
33 pilot.ti,ab,kw.
34 or/22-33
35 6 and 13 and 21 and

36 adolescent/ or child/ or child, preschool/ or exp infant/ or infant, newborn/
37 35 not 36
38 *Palliative Care/
39 37 not 38
40 LETTER/
41 EDITORIAL/
42 NEWS/
43 exp HISTORICAL ARTICLE/
44 congress.pt.
45 Anecdotes as topic/
46 Comment/
47 (letter or comment$).ti.
48 or/40-47
49 39 not 48
50 meta-analysis.pt.
51 meta-analysis/
52 systematic review/
53 Meta-Analysis as Topic/
54 "Review Literature as Topic"/
55 exp technology assessment, biomedical/
56 (systematic* adj3 review*).ti,ab,kf,kw.
57 (systematic* adj3 overview*).ti,ab,kf,kw.
58 (methodologic* adj3 review*).ti,ab,kf,kw.
59 (methodologic* adj3 overview*).ti,ab,kf,kw.
60 ((quantitative adj3 (review* or overview* or synthes*)) or (research adj3 (integrati* or overview*))).ti,ab,kf,kw.
61 ((integrative adj3 (review* or overview*)) or (collaborative adj3 (review* or overview*)) or (pool* adj3 analy*)).ti,ab,kf,kw.
62 (data synthes* or data extraction* or data abstraction*).ti,ab,kf,kw.
63 (handsearch* or hand search*).ti,ab,kf,kw.
64 (mantel haenszel or peto or der simonian or dersimonian or fixed effect* or latin square*).ti,ab,kf,kw.
65 (met analy* or metanaly* or technology assessment* or HTA or HTAs or technology overview* or technology appraisal*).ti,ab,kf,kw.
66 (meta regression* or metaregression*).ti,ab,kf,kw.
67 (meta-analy* or metaanaly* or systematic review* or biomedical technology assessment* or bio-medical technology assessment*).mp,hw.
68 (medline or cochrane or pubmed or medlars or embase or cinahl).ti,ab,hw.
69 (cochrane or (health adj2 technology assessment) or evidence report).jw.
70 (comparative adj3 (efficacy or effectiveness)).ti,ab,kf,kw.
71 (outcomes research or relative effectiveness).ti,ab,kf,kw.
72 ((indirect or indirect treatment or mixed-treatment) adj comparison*).ti,ab,kf,kw.
73 or/50-72
74 "randomized controlled trial".pt.
75 "controlled clinical trial ".pt.
76 control*.ti,ab.
77 random*.ti,ab.
78 placebo.ti,ab.
79 randomly.ti,ab.
80 trial.ti,ab.
81 groups.ti,ab.
82 or/74-81
83 exp epidemiological studies/
84 (wait-list* or waitlist* or "wait list*").ti,ab,kw.
85 (before adj3 after).ti,ab,kw.
86 (pre adj3 post).ti,ab,kw.
87 ((cohort or longintud* or prospective) and (control or comparison)).ti,ab,kw.
88 (observation* and (control or compar*)).ti,ab,kw.
89 exp comparative study/
90 exp observational study/
91 exp Longitudinal Studies/

92 exp Cohort Studies/
93 "cohort study".ti,ab,kw.
94 (baseline adj6 (week* or month* or after* or follow* or post or complet*)).ti,ab,kw.
95 exp Feasibility Studies/
96 "quasi experiment*".ti,ab,kw.
97 or/83-96
98 Economics/
99 exp cost/ and cost analysis/
100 exp economics, hospital/
101 Economics, medical/
102 Economics, nursing/
103 Economics, pharmaceutical/
104 (economic$ or cost$ or pric$ or pharmacoeconomic$).ti,ab.
105 (expenditure$ not energy).ti,ab.
106 value for money.ti,ab.
107 budget$.ti,ab.
108 or/98-107
109 73 or 82 or 97 or 108
110 49 and 109
111 exp animals/ not humans.sh.
112 110 not 111

Table S2b: PsycInfo

Search Strategy:

#	Searches	Results
1	cancer.mp. or exp Neoplasms/	64872
2	neoplasm*.mp.	45244
3	tumo?r.mp.	12122
4	malign*.mp. [mp=title, abstract, heading word, table of contents, key concepts, original title, tests & measures]	4
5	carcinoma.mp. [mp=title, abstract, heading word, table of contents, key concepts, original title, tests & measures]	1621
6	or/1-5	71255
7	exp SURVIVORS/ or survivor*.mp.	29518
8	"post treat*".mp.	7292
9	"after treat*".mp.	12147
10	(treat adj2 complet*).mp. [mp=title, abstract, heading word, table of contents, key concepts, original title, tests & measures]	67
11	aftercare.mp. or exp AFTERCARE/	2631
12	"cancer survival".mp.	326
13	survivorship.mp.	2281
14	or/7-13	50181
15	"self management".mp. or exp Self-Management/	10461
16	"self care".mp.	11266
17	"psychological adaptation".mp.	895
18	exp ADJUSTMENT/ or adjustment.mp. or exp EMOTIONAL ADJUSTMENT/	93712
19	"self concept".mp. or exp Self-Concept/	74111
20	"self efficacy".mp. or exp Self-Efficacy/	41300
21	exp Self-Help Techniques/ or "self help".mp.	15541
22	"coping skills".mp.	5350
23	"coping behavio?r".mp. or exp Coping Behavior/	45750
24	"problem solving".mp. or exp Problem Solving/	62779
25	or/15-24	322423
26	intervention.mp. or exp INTERVENTION/	249121
27	program*.mp.	386837
28	(care adj3 package).mp. [mp=title, abstract, heading word, table of contents, key concepts, original title, tests & measures]	172
29	feasability.mp.	17
30	acceptab*.mp. [mp=title, abstract, heading word, table of contents, key concepts, original title, tests & measures]	35126
31	efficacy.mp. [mp=title, abstract, heading word, table of contents, key concepts, original title, tests & measures]	136364
32	effective*.mp. [mp=title, abstract, heading word, table of contents, key concepts, original title, tests & measures]	402878
33	exp TRAINING/ or training.mp.	249641
34	educat*.mp.	550936
35	evaluat*.mp.	520257
36	exp Strategies/ or strateg*.mp.	334274
37	or/26-36	1771314
38	6 and 14 and 25 and 37	1042
39	*PEDIATRICS/	15847
40	38 not 39	1011
41	*Palliative Care/	10047
42	40 not 41	1009

Table S2c: Embase

Search Strategy:

#	Searches	Results
1	cancer.mp. or exp malignant neoplasm/	3952573
2	neoplasm*.mp. [mp=title, abstract, heading word, drug trade name, original title, device manufacturer, drug manufacturer, device trade name, keyword, floating subheading word, candidate term word]	712075
3	tumo?r.mp. [mp=title, abstract, heading word, drug trade name, original title, device manufacturer, drug manufacturer, device trade name, keyword, floating subheading word, candidate term word]	2589934
4	maligan*.mp.	427
5	exp carcinoma/ or carcinoma.mp.	1204199
6	or/1-5	4869556
7	exp survivor/ or survivor*.mp. or exp cancer survivor/	136046
8	exp post treatment survival/ or "post treat*".mp.	61482
9	(treat adj2 complet*).mp. [mp=title, abstract, heading word, drug trade name, original title, device manufacturer, drug manufacturer, device trade name, keyword, floating subheading word, candidate term word]	339
10	aftercare/	5991
11	survivorship.mp. or exp survivorship/	16026
12	"cancer survival".mp. or exp cancer survival/	300227
13	or/7-12	489099
14	"self management*.mp.	22897
15	"self care".mp. or exp self care/	78082
16	"psychological adaptation".mp.	879
17	"coping behavio?r".mp. or exp coping behavior/	57016
18	adjustment.mp. or exp adjustment/ or exp psychological adjustment/	208074
19	"self concept".mp. or exp self concept/	177251
20	"self efficacy".mp.	28311
21	"self help".mp. or exp self help/	17051
22	"coping skills".mp.	3961
23	"problem solving".mp. or exp problem solving/	40791
24	or/14-23	547525
25	intervention.mp.	795316
26	program*.mp.	1299872
27	(care adj3 package).mp. [mp=title, abstract, heading word, drug trade name, original title, device manufacturer, drug manufacturer, device trade name, keyword, floating subheading word, candidate term word]	996
28	feasability.mp. or exp feasibility study/	103380
29	acceptab*.mp.	217590
30	efficacy.mp.	1554359
31	effective*.mp.	2397177
32	training.mp. or exp training/	541401
33	exp education/ or educat*.mp.	1577564
34	exp evaluation/ or evaluat*.mp.	4538684
35	strateg*.mp.	1199524
36	or/25-35	9976057
37	6 and 13 and 24 and 36	6572
38	child/	1533886
39	infant/	541725
40	adolescent/	1393354
41	or/38-40	2580662
42	37 not 41	5797
43	*palliative therapy/	30206
44	42 not 43	5777
45	letter.pt.	1052437
46	letter/	999688
47	note.pt.	737466
48	editorial.pt.	592108
49	(letter or comment$).ti.	179259
50	or/45-49	2439325
51	44 not 50	5716

Table S2d: CINAHL

#	Query	Limiters/Expanders	Last Run Via	Results
S41	S39 NOT S40	Search modes - Boolean/Phrase	Interface - EBSCOhost Research Databases Search Screen - Advanced Search Database - CINAHL	1,605
S40	(MM "Palliative Care")	Search modes - Boolean/Phrase	Interface - EBSCOhost Research Databases Search Screen - Advanced Search Database - CINAHL	21,139
S39	S37 NOT S38	Search modes - Boolean/Phrase	Interface - EBSCOhost Research Databases Search Screen - Advanced Search Database - CINAHL	1,616
S38	(MH "Child") OR (MH "Adolescence") OR (MH "Infant") OR (MH "Infant, Newborn")	Search modes - Boolean/Phrase	Interface - EBSCOhost Research Databases Search Screen - Advanced Search Database - CINAHL	775,960
S37	S5 AND S13 AND S24 AND S36	Search modes - Boolean/Phrase	Interface - EBSCOhost Research Databases Search Screen - Advanced Search Database - CINAHL	1,891
S36	S25 OR S26 OR S27 OR S28 OR S29 OR S30 OR S31 OR S32 OR S33 OR S34 OR S35	Search modes - Boolean/Phrase	Interface - EBSCOhost Research Databases Search Screen - Advanced Search Database - CINAHL	2,264,587
S35	"strateg*"	Search modes - Boolean/Phrase	Interface - EBSCOhost Research Databases Search Screen - Advanced	224,841

S34	(MH "Evaluation+") OR "evaluat*"	Search modes - Boolean/Phrase	Interface - EBSCOhost Research Databases Search Screen - Advanced Search Database - CINAHL	953,542
S33	(MH "Education+") OR "educat*"	Search modes - Boolean/Phrase	Interface - EBSCOhost Research Databases Search Screen - Advanced Search Database - CINAHL	939,634
S32	"training"	Search modes - Boolean/Phrase	Interface - EBSCOhost Research Databases Search Screen - Advanced Search Database - CINAHL	170,349
S31	"effective*"	Search modes - Boolean/Phrase	Interface - EBSCOhost Research Databases Search Screen - Advanced Search Database - CINAHL	349,571
S30	"efficacy"	Search modes - Boolean/Phrase	Interface - EBSCOhost Research Databases Search Screen - Advanced Search Database - CINAHL	151,778
S29	"acceptab*"	Search modes - Boolean/Phrase	Interface - EBSCOhost Research Databases Search Screen - Advanced Search Database - CINAHL	37,493
S28	"feasability"	Search modes - Boolean/Phrase	Interface - EBSCOhost Research Databases Search Screen - Advanced Search Database - CINAHL	26
S27	""care N3 package""	Search modes - Boolean/Phrase	Interface - EBSCOhost Research Databases Search Screen - Advanced	675

S26	"program*"	Search modes - Boolean/Phrase	Interface - EBSCOhost Research Databases Search Screen - Advanced Search Database - CINAHL	413,981
S25	"intervention"	Search modes - Boolean/Phrase	Interface - EBSCOhost Research Databases Search Screen - Advanced Search Database - CINAHL	217,857
S24	S14 OR S15 OR S16 OR S17 OR S18 OR S19 OR S20 OR S21 OR S22 OR S23	Search modes - Boolean/Phrase	Interface - EBSCOhost Research Databases Search Screen - Advanced Search Database - CINAHL	193,461
S23	""coping behavio#r""	Search modes - Boolean/Phrase	Interface - EBSCOhost Research Databases Search Screen - Advanced Search Database - CINAHL	832
S22	(MH "Problem Solving+") OR "problem solving"	Search modes - Boolean/Phrase	Interface - EBSCOhost Research Databases Search Screen - Advanced Search Database - CINAHL	14,427
S21	"coping skills"	Search modes - Boolean/Phrase	Interface - EBSCOhost Research Databases Search Screen - Advanced Search Database - CINAHL	1,696
S20	"self help"	Search modes - Boolean/Phrase	Interface - EBSCOhost Research Databases Search Screen - Advanced Search Database - CINAHL	3,036
S19	(MH "Self-Efficacy") OR "self-efficacy"	Search modes - Boolean/Phrase	Interface - EBSCOhost Research Databases Search Screen - Advanced	23,609

S18	(MH "Self Concept+") OR "self concept"	Search modes - Boolean/Phrase	Interface - EBSCOhost Research Databases<br>Search Screen - Advanced Search<br>Database - CINAHL	54,679
S17	(MH "Social Adjustment") OR "adjustment"	Search modes - Boolean/Phrase	Interface - EBSCOhost Research Databases<br>Search Screen - Advanced Search<br>Database - CINAHL	48,551
S16	(MH "Adaptation, Psychological+") OR "psychological adaptation"	Search modes - Boolean/Phrase	Interface - EBSCOhost Research Databases<br>Search Screen - Advanced Search<br>Database - CINAHL	28,647
S15	(MH "Self Care+") OR """self care""	Search modes - Boolean/Phrase	Interface - EBSCOhost Research Databases<br>Search Screen - Advanced Search<br>Database - CINAHL	49,305
S14	""self management""	Search modes - Boolean/Phrase	Interface - EBSCOhost Research Databases<br>Search Screen - Advanced Search<br>Database - CINAHL	12,982
S13	S6 OR S7 OR S8 OR S9 OR S10 OR S11 OR S12	Search modes - Boolean/Phrase	Interface - EBSCOhost Research Databases<br>Search Screen - Advanced Search<br>Database - CINAHL	124,290
S12	"survivorship"	Search modes - Boolean/Phrase	Interface - EBSCOhost Research Databases<br>Search Screen - Advanced Search<br>Database - CINAHL	4,779
S11	(MH "After Care") OR "aftercare"	Search modes - Boolean/Phrase	Interface - EBSCOhost Research Databases<br>Search Screen - Advanced	11,827

S10	"after treatment or post treatment"	Search modes - Boolean/Phrase	Interface - EBSCOhost Research Databases<br>Search Screen - Advanced Search<br>Database - CINAHL	5
S9	""after treat*""	Search modes - Boolean/Phrase	Interface - EBSCOhost Research Databases<br>Search Screen - Advanced Search<br>Database - CINAHL	65,872
S8	""post treat*""	Search modes - Boolean/Phrase	Interface - EBSCOhost Research Databases<br>Search Screen - Advanced Search<br>Database - CINAHL	12,591
S7	(MH "Cancer Survivors") OR "cancer survivors"	Search modes - Boolean/Phrase	Interface - EBSCOhost Research Databases<br>Search Screen - Advanced Search<br>Database - CINAHL	14,251
S6	(MH "Survivors+") OR "survivors"	Search modes - Boolean/Phrase	Interface - EBSCOhost Research Databases<br>Search Screen - Advanced Search<br>Database - CINAHL	38,323
S5	S1 OR S2 OR S3 OR S4	Search modes - Boolean/Phrase	Interface - EBSCOhost Research Databases<br>Search Screen - Advanced Search<br>Database - CINAHL	595,593
S4	(MH "Carcinoma+") OR "carcinoma"	Search modes - Boolean/Phrase	Interface - EBSCOhost Research Databases<br>Search Screen - Advanced Search<br>Database - CINAHL	92,972
S3	"malignan*"	Search modes - Boolean/Phrase	Interface - EBSCOhost Research Databases<br>Search Screen - Advanced	54,227

S2	""tumo#r""	Search modes - Boolean/Phrase	Interface - EBSCOhost Research Databases<br>Search Screen - Advanced Search<br>Database - CINAHL	125,744
S1	"cancer" OR (MH "Neoplasms+")	Search modes - Boolean/Phrase	Interface - EBSCOhost Research Databases<br>Search Screen - Advanced Search<br>Database - CINAHL	545,287

Table S2e: Cochrane CENTRAL

#1	cancer* or neoplasm* or tumour* or tumor* or malignan* or carcinoma*	Limits	206670
#2	MeSH descriptor: [Neoplasms] explode all trees	MeSH	69456
#3	MeSH descriptor: [Carcinoma] explode all trees	MeSH	12009
#4	#1 or #2 or #3	Limits	212007
#5	(survivor* or "post treat*" or "after treat*" or (treat* NEAR complet*) or aftercare)	Limits	35763
#6	MeSH descriptor: [Survivors] explode all trees	MeSH	1228
#7	MeSH descriptor: [Cancer Survivors] explode all trees	MeSH	107
#8	MeSH descriptor: [Survivorship] explode all trees	MeSH	7
#9	MeSH descriptor: [Aftercare] explode all trees	MeSH	20278
#10	#5 or #6 or #7 or #8 or #9	Limits	54493
#11	#4 and #10	Limits	12193
#12	self NEXT (manage* or care or efficacy or help or guid* or direct* or regulat*)	Limits	25876
#13	#4 and #10 and #12	Limits	782
#14	intervention* or program* or (care NEAR package*) or feasab* or acceptab* or efficacy or effective* or training or educat* or evaluat* or [illegible]	Limits	955357
#15	#13 and #14	Limits	767

Table S2f: Scopus

(TITLE-ABS-KEY (cancer* OR neoplasm* OR tumour* OR tumor* OR malignan* OR carcinoma*)) AND (TITLE-ABS-KEY (survivor* OR "post treat*" OR "after treat*" OR (treat* W/2 complet*) OR aftercare)) AND (TITLE-ABS-KEY (self PRE/1 (manage* OR care OR efficacy OR help OR guid* OR direct* OR regulat*))) AND (TITLE-ABS-KEY (intervention* OR program* OR (care W/3 package*) OR feasab* OR acceptab* OR efficacy OR effective* OR training OR educat* OR evaluat* OR strateg* OR pilot))

Table S3: Protocols and papers to support intervention development

Intervention[a]	Protocol	Development paper
Chambers 2018 (Cancer Cope)	Chambers, S. K., Ritterband, L., Thorndike, F., Nielsen, L., Aitken, J. F., Clutton, S., ... & Dunn, J. (2017). A study protocol for a randomised controlled trial of an interactive web-based intervention: CancerCope. *BMJ open*, *7*(6), e017279.	NR
Foster 2016 (RESTORE)	NR	Foster, C., Calman, L., Grimmett, C., Breckons, M., Cotterell, P., Yardley, L., ... & Richardson, A. (2015). Managing fatigue after cancer treatment: development of RESTORE, a web-based resource to support self-management. *Psycho-oncology*, *24*(8), 940-949.
Frankland 2019 (Supported self-management and remote surveillance)	Frankland, J., Brodie, H., Cooke, D., Foster, C., Foster, R., Gage, H., ... & Richardson, A. (2017). Follow-up care after treatment for prostate cancer: protocol for an evaluation of a nurse-led supported self-management and remote surveillance programme. *BMC cancer*, *17*(1), 1-10.	NR
Fu 2016 (TOLF)	Fu, M. R., Axelrod, D., Guth, A., Scagliola, J., Rampertaap, K., El-Shammaa, N., ... & Melkus, G. D. E. (2016). A web-and mobile-based intervention for women treated for breast cancer to manage chronic pain and symptoms related to lymphedema: randomized clinical trial rationale and protocol. *JMIR research protocols*, *5*(1), e5104.	NR
Gregoire 2020 (Self-care and hypnosis)	Grégoire, C., Faymonville, M. E., Vanhaudenhuyse, A., Charland-Verville, V., Jerusalem, G., & Bragard, I. (2018). Randomized controlled trial of an 8-week intervention combining self-care and hypnosis for post-treatment cancer patients: study protocol. *BMC cancer*, *18*(1), 1-10.	NR
Kazer 2011 (Alive & Well)	NR	Kazer, M. W., Bailey Jr, D. E., & Whittemore, R. (2010). Out of the black box: Expansion of a theory-based intervention to self-manage the uncertainty associated with active surveillance (AS) for prostate cancer. *Research and Theory for Nursing Practice*, *24*(2), 101-112.
Kim 2021 (EMPOWER)	NR	Kim, S. H., Choe, Y. H., Han, A. R., Yeon, G. J., Lee, G. H., Lee, B. G., ... & Lee, M. H. (2020). Design of a randomized controlled trial of a partnership-based, needs-tailored self-management support intervention for post-treatment breast cancer survivors. *BMC cancer*, *20*(1), 1-9.

Intervention[a]	Protocol	Development paper
Korstjens 2008 (Self-management rehab PT and CBT)	NR	van Weert, E., Hoekstra-Weebers, J. E., May, A. M., Korstjens, I., Ros, W. J., & van der Schans, C. P. (2008). The development of an evidence-based physical self-management rehabilitation programme for cancer survivors. *Patient education and counseling*, *71*(2), 169-190.
Krouse 2016 (OSMT)	NR	Grant, M., McCorkle, R., Hornbrook, M. C., Wendel, C. S., & Krouse, R. (2013). Development of a chronic care ostomy self-management program. *Journal of Cancer Education*, *28*(1), 70-78.
Lee 2014 (WSEDI)	NR	Lee, M. K., Park, H. A., Yun, Y. H., & Chang, Y. J. (2013). Development and formative evaluation of a web-based self-management exercise and diet intervention program with tailored motivation and action planning for cancer survivors. *JMIR research protocols*, *2*(1), e2331.
Meneses 2017 (Support and early educational telehealth)	NR	Meneses, K., Gisiger-Camata, S., Schoenberger, Y. M., Weech-Maldonado, R., & McNees, P. (2015). Adapting an evidence-based survivorship intervention for Latina breast cancer survivors. *Women's Health*, *11*(2), 109-119.
Skolarus 2019 (Building your new normal)	Skolarus, T. A., Metreger, T., Hwang, S., Kim, H. M., Grubb, R. L., Gingrich, J. R., & Hawley, S. T. (2017). Optimizing veteran-centered prostate cancer survivorship care: study protocol for a randomized controlled trial. *Trials*, *18*(1), 1-14.	NR
Turner 2019 (ENHANCES)	Turner, J., Yates, P., Kenny, L., Gordon, L. G., Burmeister, B., Thomson, D., ... & Carswell, K. (2014). The ENHANCES study—Enhancing Head and Neck Cancer patients' Experiences of Survivorship: study protocol for a randomized controlled trial. *Trials*, *15*(1), 1-9.	NR
Van den Berg 2015 (BREATH)	van den Berg, S. W., Gielissen, M. F., Ottevanger, P. B., & Prins, J. B. (2012). Rationale of the BREAst cancer e-healTH [BREATH] multicentre randomised controlled trial: an internet-based self-management intervention to foster adjustment after curative breast cancer by decreasing distress and increasing empowerment. *BMC cancer*, *12*(1), 1-13.	NR
Van der Hout 2020 (Oncokompas)	Van Der Hout, A., van Uden-Kraan, C. F., Witte, B. I., Coupé, V. M., Jansen, F., Leemans, C. R., ... & Verdonck-de Leeuw, I. M. (2017). Efficacy, cost-utility and reach of an eHealth self-management application 'Oncokompas' that helps cancer survivors to obtain optimal supportive care: study protocol	Duman-Lubberding, S., van Uden-Kraan, C. F., Peek, N., Cuijpers, P., Leemans, C. R., & Verdonck-de Leeuw, I. M. (2015). An eHealth application in head and neck cancer survivorship care: health care professionals' perspectives. *Journal of medical Internet research*, *17*(10), e4870.

Intervention[a]	Protocol	Development paper
	for a randomised controlled trial. *Trials*, *18*(1), 1-11.	
Watson 2018 (PROSPECTIV)	Watson, E., Rose, P., Frith, E., Hamdy, F., Neal, D., Kastner, C., ... & Matthews, M. (2014). PROSPECTIV—a pilot trial of a nurse-led psychoeducational intervention delivered in primary care to prostate cancer survivors: study protocol for a randomised controlled trial. *BMJ open*, *4*(5), e005186.	NR
Willems 2016 (KNW)	Willems, R. A., Bolman, C. A., Mesters, I., Kanera, I. M., Beaulen, A. A., & Lechner, L. (2015). The Kanker Nazorg Wijzer (Cancer Aftercare Guide) protocol: the systematic development of a web-based computer tailored intervention providing psychosocial and lifestyle support for cancer survivors. *BMC cancer*, *15*(1), 1-16.	NR

[a]Though this review included 32 interventions, this table only reports those with a published protocol or development paper.
NR = not reported

Table S4: Additional population characteristics of cancer survivors

Study	Eligible population	Ethnicity	Stage of cancer
Chambers 2018	Adults diagnosed with cancer, score ≥4 on the Distress Thermometer (indicating high distress or risk of high distress)	NR	NR
Faithfull 2010	Locally confined prostate cancer (stage T3bNO or less), >3 months post completion of radiotherapy, received neo-adjuvant hormone therapy, and have moderate to severe urinary symptoms (IPSS score ≥8)	NR	NR
Foster 2016	Adults (≥18 years old) with invasive cancer ≤5 year previously, completed curative intent treatment, without metastatic disease, self-reported moderate to severe fatigue	I: White (97.6%), Non-white (2.4%);<br>C: White (100%)	NR
Frankland 2019	Prostate cancer patients within 3 years of completion of radical prostatectomy or radiotherapy or within 3 years of commencement of primary androgen deprivation therapy	I: White (97%), Mixed (0%), Asian (1%), Black (1%), Other (1%);<br>C: White (99%), Other (1%)	I: Stage I (11%), II (42%), III (42%), IV (4%);<br>C: Stage I (11%), II (45%), III (39%), IV (4%)
Fu 2016	Breast cancer patients ≥21 years of age who had surgical treatment (lumpectomy or mastectomy, sentinel lymph node biopsy or axillary lymph node dissection, with or without been diagnosed or treated for lymphedema.	I: White (65%), Asian (10%), African American/ Black (10%), Hispanic/Latino (10%), Arabic (5%)	NR
Gregoire 2020, Gregoire 2021	Adults (≥18 years old), fluent in French, presenting with a non-metastatic invasive cancer, completed active treatments since less than a year, and experience baseline difficulties (score of at least 4 out of	NR	NR

Study	Eligible population	Ethnicity	Stage of cancer
	10 on 1 of these 6 items: physical fatigue, moral fatigue, depression, anxiety, fear of recurrence, ruminations)		
Kazer 2011	Men diagnosed with prostate cancer and currently undergoing active surveillance	I: White (100%)	NR
Kim 2021	Women aged ≥19 years, breast cancer diagnosis, treated with a curative cancer therapy, completed primary cancer treatment within previous 6 months, two or more unmet needs, able to use the telephone. Excluded if they had a recurrence, metastasis or another cancer	NR	I: Stage 0 or I (46.8%), II (36.2%), III (17%);<br>C: Stage 0 or I (48.9%), II (29.8%), III (21.3%)
Korstjens 2008, Korstjens 2011, May 2008, May 2009, van Weert 2010	All cancers, age ≥18 years, last curative cancer treatment completed at least 3 months before study entry, estimated life expectancy >1 year.	NR	NR
Krouse 2016, Hornbrook 2018, Cidav 2021	All cancer survivors with ostomies	I: White (84.2%), Hispanic (2.6%), Black (2.6%)	NR
Kvale 2016	Female breast cancer, inclusion: age ≥19 years; diagnosed with non-metastatic cancer (AJCC TNM stage 0- IIIb); and within 1 year of completing active cancer treatment.	I: Non-Hispanic white (82.5%), African American (12.5%), Asian (2.5%), Other (2.5%);<br>C: Non-Hispanic white (79.5%), African American (20.5%)	NR
Lawn 2015, Miller 2016	Men and women diagnosed with solid tumours, treated with curative intent	I: Caucasian (93%), Asian (7%);<br>C: Caucasian (91%), Other (9%)	NR
Lee 2010	Adults with a gastric cancer diagnosis after gastrectomy	NR	I: Stage I and II
Lee 2014	Breast cancer, female, stage 0-III disease, who had undergone curative surgery and completed primary treatment within past 12 months	NR	I: Stage 0 (6.7%), I (40%), II (43.3%), III (10%);<br>C: Stage I (37.9%), II (51.7%), III (10.3%)
Loubani 2021	Women with invasive ductal carcinoma, stages I to III, 3-24 months post breast cancer diagnosis, completed primary adjuvant therapies, previously healthy, reported difficulties or decreased participation in daily activities. Exclusion if severe disability that affected daily functioning.	NR	I: Stage I (11.1%), II (55.6%), III (33.3%);<br>C: Stage I (35.3%), II (29.4%), III (35.3%)
Manne 2020	Age >18 years, diagnosed with a first primary oral or oropharyngeal cancer 1-3 years ago, currently cancer free, computer access, can read English	I: Non-Hispanic White (86.4%), Minority (10.6%), missing (3%)	I: Localised (25.8%), regional (51.5%)
Mardani 2020	Prostate cancer diagnosis, active participation in treatment sessions within the past 3-12 months, no metastasis, no history of disorders	NR	NR

Study	Eligible population	Ethnicity	Stage of cancer
	affecting physical activity level, age >45, living in an urban area. Exclusion if unwilling to take part and presence of any health issue that hindered participation in physical activity		
McCusker 2021	Aged ≥18 years, completed primary treatment for any cancer type, diagnosed 1-10 years previously, in remission, PHQ-9 score of 8-19 indicating mild to moderate depression. Exclusion if metastatic disease, nonmelanoma skin cancer, suicidal intent, moderate-severe cognitive impairment, still receiving treatment, started or adjusted antidepressant medication in previous 6 weeks, and unable to speak/read English/French.	NR	NR
Meneses 2017[a]	Latina breast cancer survivors ≥21 years old with Stage 0–III breast cancer, within the first 3 years of completing primary breast cancer treatment	I: Hispanic/Latina (100%)	Stage 0-III
Moon 2019	Women, (≥18 years old), had a diagnosis of primary breast cancer, had been prescribed adjuvant tamoxifen and had suboptimal levels of adherence, as evidenced by scoring <25 on the Medication Adherence Rating Scale	I: White British (79%), Other (18%)	I: Stage I (39%), II (42%), III (18%)
Newman 2019	Women (≥18 years old) who were diagnosed with stage 0–III breast cancer, had completed chemotherapy, hormone therapy, radiation or surgery 6 months to 2 years previously	I: Black (74%), White (13%), Other (13%)	I: Stage 0 (13%), I (33%), II (47%), III (7%)
Omidi 2020	A history of confirmed breast cancer (stages 0 to IV), lymphedema established by a physician in the past year, aged 18–65 years old, completion of primary cancer treatments, ability to read and write and work with the Telegram™ messenger, no post-cancer psychiatric disorders requiring drug therapy, and access to the internet through cell phones or computers. Exclusion if fail to attend in the third and fourth sessions of in-person education as the key sessions, failure to approve delivery of messages in the Telegram™ in the SNE group, detection of cancer recurrence during the study, and unwillingness to continue the intervention.	NR	I: GE: Stage I (6.2%), II (46.9%), III/IV (46.9%); I: SNE: Stage I (5.9%), II (64.7%), III/IV (29.4%); C: Stage I (6.4%), II (61.3%), III/IV (32.3%)
Salvatore 2015, Ahn 2013, Ory 2013[b]	At least one self-reported chronic condition or disease	I: Non-Hispanic white (75%), African American (7.8%), Latino/Hispanic (10.3%), Asian (3.5%), American Indian (0.9%), Other (2.6%); C: Non-Hispanic white	NR

Study	Eligible population	Ethnicity	Stage of cancer
		(53%), African American (16.9%), Latino/Hispanic (23.6%), Asian (2.9%), American Indian (0.7%), Other (3%)	
Schmidt 2016	≥14 years old, received allogeneic or autologous HSCT as part of their cancer treatment	NR	NR
Skolarus 2019	Prostate cancer, 40 to 80 years old, not undergoing treatment for a separate cancer	I: White (71.2%), Black (26.6%), Other (3.2%); C: White (66.7%), Black (29.9%), Other (5.4%)	NR
Turner 2019	Age ≥ 18 years, completed within the past month curative intent definitive treatment (surgery, radiotherapy, chemotherapy, or a combination of these) for cancer of the tongue, mouth, salivary glands, pharynx, oro-, hypo-, and nasopharynx, nasal cavities, middle ear, sinuses, or larynx; or completed a defined treatment protocol for non-melanoma skin cancers of the head and neck requiring radiotherapy, chemotherapy, or surgery.	NR	NR
Van den Berg 2015, Van den Berg 2013	Breast cancer, histologically confirmed and completed curative intent treatment 2-4 months before baseline assessment	NR	NR
Van der Hout 2020a, Van der Hout 2020b, Van der Hout 2021a, Van der Hout 2021b, Duman-Lubberding 2016	Age ≥18 years, head and neck, colorectal, breast cancer or Hodgkin lymphoma or NHL, and between 3 months and 5 years after treatment with curative intent	NR	I: Stage I (n=106), II (n=73), III (n=61), IV (n=64); C: Stage I (n=104), II (n=70), III (n=67), IV (n=52)
Watson 2018, Burns 2017	Prostate cancer, stable, treated by surgery, radiotherapy, ADT or active surveillance; self-reported ongoing problem with urinary, bowel, sexual or hormone-related functioning	I: White British (97.6%), White other (2.4%); C: White British (95.1%), White other (2.4%), Other (2.4%)	NR
Willems 2016, Willems 2017a, Willems 2017b, Kanera 2016a, Kanera 2016b, Kanera 2017	All cancers, Dutch speaking, aged ≥18, completed primary treatment (surgery, chemo, radio) 4-56 weeks previously	NR	NR
Yun 2012	Age 20-65 years, moderate to severe fatigue (worst fatigue in Brief Fatigue Inventory ≥4) for at least 1 week, cancer stages I to III, primary treatment completed within the past 24 months	NR	I: Stage I (n=80), II (n=36), III (n=20); C: Stage I (n=65), II (n=48), III (n=24)
Zhang 2015	Early stage (I, II or III) prostate cancer, completion of cancer treatment for at least 6 months and presenting incontinent symptoms	I: White (61.3%), Black (38.8%); I: T: White (66.3%), Black	I: Stage I and II (n=76), III (n=4); I: T: Stage I and II (n =

Study	Eligible population	Ethnicity	Stage of cancer
		(32.5%), Other (1.3%);<br>C: White (63.8%), Black (35%), Other (1.3%)	76), III (n = 5)<br>C: Stage I and II (n = 78), III (n = 4)

[a]Population characteristics were not reported separately for intervention and control groups.
[b]Population characteristics were not reported separately for cancer survivors within the intervention group.
C = comparator arm; GE = group education; I = Intervention arm; NR = Not Reported; SNE = social network education; T = telephone support

Table S5: Theory and rationale for the intervention

Study	Theory and rationale
Chambers 2018	Based on CBT.
Faithfull 2010	No theory stated; self-management can offer the means to ameliorate the effects of prostate cancer and its treatment.
Foster 2016	Foster & Fenlon framework for recovery of health and wellbeing after cancer treatment which assumes that cancer and its treatment disrupt people's lives, and requires a process of recovery. The framework is underpinned by Bandura’s self-efficacy theory and predicts that with appropriate support, cancer survivors can self-manage problems related to cancer and its treatment. Including behaviour techniques will enhance self-efficacy to self-manage the impact of cancer-related fatigue on everyday life. Intervention also informed by CBT.
Frankland 2019	Based on the key principles of risk stratification, individualised tailored care and support, formalised care planning, remote surveillance, rapid access to clinic when appropriate and empowerment to self-manage through enhancement of knowledge, skills and confidence in order to provide person-centred care through which to address men’s survivorship needs. Workshops were informed by Bandura's social cognitive theory, Adair's action-centred leadership models and principles of andragogy.
Fu 2016	Development guided by Model of Self-Care for Lymphedema Symptom Management (based on author's previous research) and health habit formation; intervention focuses on building self-care skills based on research-based, innovative, safe, feasible and easily-integrated-into-daily-routine self-care strategies to lessen lymphedema symptom burden.
Gregoire 2020, Gregoire 2021	Self-care approach is based on self-management and patient empowerment approaches which aim to strengthen self-esteem, assertion and self-confidence. States some strategies used in intervention are similar to those developed in CBT. Hypnosis defined as ‘a procedure during which a health professional/researcher suggests that a patient experience changes in sensations, perceptions, thoughts or behaviour’. It is expected that self-hypnosis will influence cognition and emotional regulation and facilitate the completion of the assigned tasks. Hypothesis is that intervention would impact fatigue through its impact on other factors such as emotional distress.
Kazer 2011	Mishel's Uncertainty in Illness Theory; self-efficacy. By improving self-efficacy (via self-management of cancer via diet, exercise, smoking cessation, alcohol moderation, rest), a patient's sense of control over the cancer would be increased, and uncertainty would be decreased.
Kim 2021	Based on the CCM that emphasises that a productive interaction between patients and their practice team is essential to good care. By improving interactions, health outcomes are improved. This interaction facilitated via the partnership-based properties of the intervention. Also based on conceptual self-management framework and skills of Lorig and Holman.
Korstjens 2008, Korstjens 2011, May 2008, May 2009, van Weert 2010	Both the PT and CBT components were based on principles of self-management. Guiding theories for PT component was application of physiological training principles; self-management (Self-regulation theory); self-efficacy (Social Cognitive Theory); and illness perceptions (Common Sense model). By adding CBT to PT, it may positively influence physical activity by increasing self-efficacy and decreasing perceived barriers to exercise. Rational perceptions considered a pre-requisite for active coping/behavioural change. CBT component was aimed at training self-management skills based on cognitive-behavioural problem-solving approach of Nezu. CBT aimed to enable participants to effectively solve their personal problems associated with cancer.

Study	Theory and rationale
Krouse 2016, Hornbrook 2018, Cidav 2021	The CCM was the overarching theoretical framework. Self-management an essential component of the model in which partnerships are formed between patients and providers. These partnerships help to empower patients so they can ultimately manage their own care. Self-management education to enhance self-efficacy, teaches problem solving skills and cognitive restructuring (based on Chronic Disease Self-Management Program by Lorig). Self-efficacy is enhanced when patients succeed in problem solving.
Kvale 2016	Intervention is framed on the CCM and incorporates elements of the CTIM by Coleman. The CCM posits that for patients to manage their chronic illness, they must set goals and establish a plan to improve their health, and engender the motivation, information, skills, and confidence necessary to manage their illness. The CTIM intended to provide patients with support for self-management and increase patient activation.
Lawn 2015, Miller 2016	An evidence-based, structured interview processes using cognitive behavioural and motivational processes that allowed for assessment of self-management behaviours, enablers and barriers to change, and collaborative identification of problems and goals, leading to the development of an individualised person-centred self-management care plan.
Lee 2010	Principles of self-help management.
Lee 2014	Transtheoretical model. By increasing the stage of change, enhancing self-efficacy and encouraging the process of change, it can create behavioural changes that lead to improvements in health-related QoL, depression, anxiety and fatigue.
Loubani 2021	Intervention adopts the Model of Human Occupation as overarching theoretical basis to understand how women after breast cancer experience disruptions to their occupational performance and participation, their motivations and what adaptions are needed. Practically, principles for intervention are drawn from 1) self-management strategies (e.g. solving problems); 2) biomechanical approach (e.g. environmental approach) and 3) multi-context treatment approach to train metacognitive strategies related to daily activities.
Manne 2020	Social cognitive theory and the development of self-efficacy. Content guided by several other relevant constructs: 1) Gollwitzer and Schwarzer's work on planning as key component of self-management; 2) construct of activation defined as the willingness to take actions to manage care and understanding one's role in survivorship care; 3) knowledge of tasks to improve self-efficacy; 4) support needs which are linked to self-efficacy.
Mardani 2020	SMA and social cognitive theory. SMA offers a strategy to reduce the effects of prostate cancer and its treatment through prioritising men's healthcare needs and boosting their motivation for participation in healthcare.
McCusker 2021	Adapted for cancer survivors from an evidence-based telephone-supported depression supported self-care intervention for primary care patients with chronic physical conditions. Toolkit incorporates principles of, and exercises using CBT.
Meneses 2017	No theory stated; linguistic and culturally based interventions can ameliorate problems and improve QoL for Latinas.
Moon 2019	Development informed by CBT, the Common-Sense Model of Illness Theory or Planned Behaviour. Aimed to modify known determinants of tamoxifen non-adherence including key beliefs about cancer recurrence and tamoxifen use, management of side-effects and increase perceived behavioural control over medication taking.
Newman 2019	Three specific psychological theoretical frameworks were incorporated in the development of this intervention: self-efficacy theory (Bandura); social cognitive theory (Bandura) and problem-solving therapy (D'Zurilla and Nezu), a form of CBT. The Person Environment Occupational Model (Law) was also included in the design of the intervention to move beyond symptom management and promote a client-centred approach to maximizing occupational performance.
Omidi 2020	No theory stated; educational content was designed after a review of the literature on lymphedema self-management behaviours.

Study	Theory and rationale
Salvatore 2015, Ahn 2013, Ory 2013	Program based on self-efficacy theory and is designed to enhance personal efficacy through skills mastery, reinterpretation of symptoms, modelling and social persuasion.
Schmidt 2016	Intervention aims to increase patient participation and to improve self-management abilities by means of self-assessment and active involvement in treatment measures.
Skolarus 2019	Based on the conceptual framework of 'Self-Management and Recovery following Cancer Treatment' proposed by Foster and Fenlon. The theoretical foundations for this framework is (e.g. the role of self-efficacy in health and wellbeing) and the transactional model of stress and coping (e.g. coping appraisal). Intervention uses a patient empowerment approach to specifically improve coping appraisal, self-efficacy for interacting with clinicians, and self-management strategies thereby potentially improving subjective health and cancer outlook. Intervention also incorporates elements of CBT-based self-management. CBT strategies are particularly relevant because they are specifically designed to simultaneously target reductions in symptoms as well as associated disability, emotional distress, and QoL.
Turner 2019	Informed by the principles contained in the chronic condition and self-management model. Promotion of self-efficacy is the fundamental theoretical platform of intervention. Targeting self-efficacy is likely to lead to sustained improvements in self-management of ongoing health concerns, with the improvements lasting beyond the duration of the intervention.
Van den Berg 2015, Van den Berg 2013	Program based on cognitive behavioural therapy which provides participants with self-management skills to enable them to take control of, and adjust to, post-treatment survivorship. In concordance with the stress-coping model of Leventhal, emotional well-being after cancer is a balance between stress and resources. To target these to aspects of well-being, the intervention aims to decrease psychological stress and increase psychological empowerment. Also developed using the transactional model of strass and the model of psychological well-being in cancer survivors.
Van der Hout 2020a, Van der Hout 2020b, Van der Hout 2021a, Van der Hout 2021b, Duman-Lubberding 2016	Based on the CCM for self-management.
Watson 2018, Burns 2017	Based on a self-management approach and underpinned by Bandura's Social Cognitive Theory. Through self-reflection people make sense of their experiences, explore their own cognitions and self-beliefs, engage in self-evaluation and alter their thinking and behaviour accordingly. Self-efficacy is central to wellbeing. Unless people believe their actions can produce desired outcomes there is little incentive to act.
Willems 2016, Willems 2017a, Willems 2017b, Kanera 2016a, Kanera 2016b, Kanera 2017	Principles of problem-solving therapy, cognitive behavioural therapy, social cognitive behaviour change theories and self-regulation theories (e.g. Theory of Planned Behaviour, Self-regulation Theory, Integrated Model for Change) were applied through modules.
Yun 2012	Areas of intervention were based on the transtheoretical model of health behaviour change and social cognitive theory as developed by Bandura or on CBT.
Zhang 2015	No theory stated; intervention adopted a patient centred approach which would enable patient activation and engagement.

CBT = Cognitive behavioural therapy; CCM = Chronic care model; CTIM = Care transitions interventions model; PT = Physical therapy; QoL = Quality of life; SMA = Self-management approach

Table S6: Lorig and Holman self-management tasks

Study	Medical management	Role management	Emotional management
Chambers 2018	Unclear; not clear if Core 5 which included tips for improving sleep and fatigue covered medical management.	No	Yes; core components covered relaxation, how to cope with stressful situations, understanding stress and managing worry.
Faithfull 2010	Yes; cognitive component - recognising urinary problems. Behavioural component - self-monitoring of symptoms, bladder retraining exercises, pelvic floor exercises.	No	Yes; cognitive component – emotional support
Foster 2016	Yes; session 1 focused on an introduction to CRF - what it is, its causes, effects. Provision of strategies to manage/cope with CRF throughout sessions including via exercise, diet, sleep.	Yes; session 3 focused on work and home life. Session describes how CRF may impact on these aspects of everyday life and how effective goal setting can help to manage this interference. Session 5 focused on talking to others and strategies to manage this (includes employers, families, friends, healthcare professionals, others for information/support).	Yes; session 4 focused on managing thoughts and feelings (includes self-esteem, uncertainty, worry/anxiety, negative thoughts and feelings, stress/relaxation).
Frankland 2019	Yes; Patient Online System prompts users when blood tests are due; workshop included information on symptom management, when to consult clinical team.	No	Yes; workshop included relaxation techniques.
Fu 2016	Yes; information given on lymphedema self-care and exercises to promote lymph flow and mobility.	No	No
Gregoire 2020, Gregoire 2021	No	Unclear; states examples of assignments were 'adjusting self-expectation', 'adaption of social roles', 'findings one's own boundaries and personal needs', but not specified.	Yes; the 8 sessions and assigned tasks covered a variety of issues such as importance of pleasing ourselves every day, mental imagery exercises, paying attention to small successes, importance of congratulating self, relaxation exercises, managing rumination.
Kazer 2011	Unclear; states information given about prostate cancer and active surveillance but not specified.	Unclear; states email-based intervention probed for problems in areas including broader life issues (e.g., relationships with spouse and family), but not specified.	Yes; internet intervention included cognitive reframing such as avoiding negative thoughts and focusing on the positive and normalising. The email-based intervention probed for self-care management concerns but not specified.
Kim 2021	Yes; education session 1 focused on medical management - follow-up after treatment, long term/late effects, sign/symptoms of recurrence, second cancer screening, vaccination, side effects of anti-hormone therapy. Session 2 focused on symptom management - pain, fatigue, insomnia, lymphedema, peripheral neuropathy.	Yes; education session 5 focused on body image, sexuality, return to work, recovery of self-confidence.	Yes; education session 4 focused on emotional management – distress.
Korstjens 2008, Korstjens 2011, May	*PT only intervention arm:* Yes, individual PT sessions tailored	*PT only intervention arm:* Unclear, states role functioning were taught	*PT only intervention arm:* No. *PT and CBT intervention arm:* Yes;

Study	Medical management	Role management	Emotional management
2008, May 2009, van Weert 2010	exercise to individuals capabilities. *PT and CBT intervention arm:* Yes; as above and CBT sessions involved psychoeducation about fatigue, exercise physiology.	how to restore the balance between demand and capacity during tasks and activities but not specified. *PT and CBT intervention arm:* Yes; as above but in CBT sessions, participants learned to apply self-management skills in striving for personal goals (e.g. in work, household, hobbies, physical activity, family relationships and social contacts).	CBT sessions involved psycho-education on stress, relaxation, promoting optimism.
Krouse 2016, Hornbrook 2018, Cidav 2021	Yes; sessions covered definitions, associated disease states, potential ostomy complications, monitoring ostomy output, daily care, skin care, clothing changes, practice with equipment.	Yes; sessions covered social well-being, problems of social/ interpersonal relationships, public appearances, being prepared for emergencies, intimacy and sexuality, and communication skills within the cultural framework of the individual participant and their family in home and social contexts.	Yes; sessions covered impact on feelings, improving psychological health and attitudes.
Kvale 2016	Yes; review of survivorship care plan, review of health care team, discussion of symptom management.	No	No
Lawn 2015, Miller 2016	Unclear; not explicitly stated although a care plan was developed and provided to participant.	No	No
Lee 2010	No	Unclear; self-help education classes stated to provide information on activity of daily life management but not specified.	Yes; self-help educations sessions provided information on emotional management.
Lee 2014	No	No	No
Loubani 2021	No	Yes; intervention aimed to improve women's participation in meaningful daily activities. Sessions involved training of motor and cognitive strategies which can be transferred into daily activities at home/work/community environment.	No
Manne 2020	Yes; all four modules addressed aspects of medical management - managing dry mouth, dental hygiene, swallowing difficulties, conducting an oral self-exam, managing mouth pain, recommended long-term follow-up care.	No	No
Mardani 2020	Yes; exercise programme aimed to help with reduction of physical complications of prostate cancer.	No	No
McCusker 2021	Yes; Toolkit contained information on life after cancer treatment including side effects, information on depression and depression treatment. A medication and medical appointment tracker.	No	Yes; Toolkit contained information on mood, positive coping. Contained links for audio/video files to learn to practice relaxation techniques and information on exercises about managing worry. Included a mood monitoring tool.

Study	Medical management	Role management	Emotional management
Meneses 2017	Yes; sessions 1 and 2 covered physical complaints and changes resulting from cancer and its treatment and symptom management. Telephone sessions also stated to cover symptom management.	Unclear; stated that financial, social and family impact of cancer addressed.	Yes; 3rd session addressed the emotional and spiritual impact of breast cancer diagnosis and treatment (e.g. anxiety, fear of recurrence, meaning in illness, and the social and family impact).
Moon 2019	Yes; section 1-3 covered what is tamoxifen, how to take tamoxifen and side effects of tamoxifen.	No	No
Newman 2019	Yes; content included occupational therapy management of modifiable factors that contribute to cancer-related cognitive impairment such sleep dysfunction, fatigue, pain.	Yes; problem-solving and group brainstorming sessions to generate potential solutions to occupational performance challenges across self-care, work and leisure and social participation and also weekly action planning for self-selected occupational performance challenges. Session 4-5 covered work and productive activities, and leisure and social participation.	Yes; content on emotional distress.
Omidi 2020	Yes; Group education arm: educational content on lymphedema self-management education and skills training. Not specified for social network-based education group, but suggested to be similar to that of group education arm.	No	Yes; educational content on stress management strategies.
Salvatore 2015, Ahn 2013, Ory 2013	Yes; states programme aimed to develop skills necessary for medical management of chronic conditions including using medications. However, not designed specifically for cancer survivors.	Yes; states programme aimed to develop skills necessary for role management of chronic conditions. However, not designed specifically for cancer survivors.	Yes; states programme aimed to develop skills necessary for emotional management of chronic conditions. Topics included relaxation/cognitive symptom management, anger/fear/frustration, depression. However, not designed specifically for cancer survivors.
Schmidt 2016	Yes; included increasing patients participation in oral management including a mouth-care protocol describing their tasks in the mouth-care regime. Also exercise training to reduce muscle weakness.	No	Yes; included progressive muscular relaxation techniques if participants felt the need for relaxation.
Skolarus 2019	Yes; automated phone calls enabled participants to identify a priority symptom. Personalised newsletters then provided information regarding their symptoms and strategies to help them manage them. Included self-management, pharmacologic, medical, and surgical management strategies.	No	No
Turner 2019	*Intervention arm:* Yes; patients received a written copy of a tailored HNCP. Patients encouraged to engage with their general practitioner for ongoing support in implementing	*Intervention and information only arms*: Yes; both received written 'Facing the Future' booklet which provided information on work, day-to-day tasks, relationships and social	*Intervention and information only arms:* Unclear; states that relaxation and other techniques are used in the study to reduce physiological

Study	Medical management	Role management	Emotional management
	HNCP. Session provided information on/exploration of patient's symptom management/ongoing health concerns. Also received a copy of 'Facing the Future' but unclear if resource included medical management. *Information only arm:* Unclear; patients received a copy of 'Facing the Future' but unclear if this resource included medical management.	functioning. Unclear if role management covered in individual supportive/educational sessions and HNCP of Intervention arm as these were based on patient's individual concerns/health needs.	arousal/promote self-efficacy, but no further information given.
Van den Berg 2015, Van den Berg 2013	Yes; all phases contained information about breast cancer/it's treatment, its consequences and dealing with consequences.	Yes; phase 3 covered return to work and talking about breast cancer at work. May be covered in other phases but not clear.	Yes; all phases included emotional management e.g. emotional effects of breast cancer/it's treatment, personal grief, fear of recurrence, coping with fear, relaxation.
Van der Hout 2020a, Van der Hout 2020b, Van der Hout 2021a, Van der Hout 2021b, Duman-Lubberding 2016	Yes; tumour specific information (e.g. for head and neck cancer); unclear for 2020 version onwards	Yes; social quality of life topic covers return to work, relationship with partner/children.	Yes; psychological quality of life topic covers anxiety, depression, fear of recurrence, stress, subjective cognitive functioning.
Watson 2018, Burns 2017	Yes; appointments aimed to deliver best available evidence for management of urinary, bowel, sexual and hormonal functioning problems. Depending on participants needs they could be offered advice on pelvic floor exercises, bladder retraining or drugs for erectile dysfunction. Further referral (e.g. to GP) if appropriate.	No	Yes; initial appointments aimed to explore concerns and anxieties; follow-up appointments also aimed to identify specific situations/thought patterns that caused distress.
Willems 2016, Willems 2017a, Willems 2017b, Kanera 2016a, Kanera 2016b, Kanera 2017	Yes; module on managing cancer-related fatigue which includes use of exercise and diet. Participants are given advice to deal with symptoms (e.g. pain, lymphedema) but are advised to contact their physician or other health professional when they experience serious problems.	Yes; module on return to work which covers difficulties in returning to work and has themes of Communication (preparing for difficult work-related conversations), Balance (finding a balance between work abilities and their workload), and Rights and Obligations (provides information on cancer survivors rights and obligations concerning work with a long-term illness). Relationships module addressing coping with difficult social situations and intimacy problems.	Yes; module on mood. Also principles of CBT are addressed within modules including monitoring behaviours and thoughts and relaxation exercises.
Yun 2012	Yes; included a general introduction to cancer-related fatigue, energy conservation, physical activity, nutrition, sleep hygiene, pain control.	No	Yes; distress management.
Zhang 2015	Yes; all participants attended biofeedback session and were taught pelvic floor muscle exercises. Problem-solving therapy sessions could involvement management of consumption on noncaffeinated fluid,	No	No

Study	Medical management	Role management	Emotional management
	setting bladder voiding schedules, maintaining at diet to avoid constipation and performing daily exercise.		

CBT = Cognitive behavioural therapy; CRF = Cancer related fatigue; HNCP = Head and neck cancer survivor self-management care plan; PT = Physical therapy

Table S7: Implementation issues

Study	Recruitment rate	Reasons for non-participation	Intervention adherence	Reasons for withdrawal	Intervention modifications	Fidelity to the protocol
Chambers 2018	11.3% (163/1443) consented and eligible/contacted	Did not respond (n=601); refused (n=302); not eligible (n=377)	10% (8/79) accessed all 6 'cores', 28% (22/79) accessed 3 or more 'cores' (and were classified as having completed the intervention), 47% (37/79) did not access any 'cores'. Intervention arm adherence: 44 completed outcome and process evaluation questionnaires; 27 did not; 8 patients were withdrawn. Additional factors affecting adherence related to the internet-based nature of the intervention are also reported.	Not interested (n=3); access issues (n=2); required alternative support (n=1); no longer eligible (n=1); doesn't need support (n=1).	NR	Authors report that they were unable to recruit their target number of patients.
Faithfull 2010	31% (22/71) eligible and consented/ assessed for eligibility	Not eligible (n=28), declined (n=21: parking costs, co-morbidities, restricted mobility)	32% (7/22) dropped out during programme	Death (n=1); travel commitments (n=1); reason not given (n=3); co-morbidity (n=1); failed to submit final questionnaires (n=1)	NR	NR
Foster 2016	15.8% (163/1032) randomised/assessed for eligibility	Not eligible (n=586); not interested (n=15); not computer literate (n=10); too busy (n=3); did not understand study (n=1); fatigue not cancer related (n=13); no reason given (n=1); did not follow up with research team (n=151); did not respond to contacts (n=21); did not wish to take part (n=25); no longer eligible (n=15); did not register online (n=24); did not complete T0	43% logged on to all 5 sessions; 71% logged on to 3 sessions and, as such, were considered to have adhered to the intervention	Active withdrawal (n=7); no further contact for some or all data collection (n=25)	NR	Researchers were unable to prevent the intervention arm from seeing the publicly available leaflet that they intended to be only accessed by the control arm.

Study	Recruitment rate	Reasons for non-participation	Intervention adherence	Reasons for withdrawal	Intervention modifications	Fidelity to the protocol
		questionnaire (n=3); incorrectly identified as eligible (n=1)				
Frankland 2019	63.3% (304/480) consented/ assessed for eligibility	Did not respond (n=121), declined invitation (n=38), ineligible (n=8), response too late (n=4), missed by researcher (n=3), pack not sent as refused workshop (n=2)	NR	NR	NR	NR
Fu 2016	NR	NR	NR	NR	The programme was iteratively refined in the usability evaluation phase.	NR
Gregoire 2020, Gregoire 2021	91.2% (104/114) randomised/eligible	Declined to participate (n=10)	Average attendance was 6.48 sessions out of 8. Eight participants dropped out between T1 and T2.	NR	NR	NR
Kazer 2011	NR	NR	Men viewed the webpages 2-40 times (average 20 page views)	NR	NR	NR
Kim 2021	67.1% (94/140) randomised/assessed for eligibility	Declined to participate (n=28), not eligible: other cancer (n=1), recurrence (n=1), cancellation of treatment (n=2), undergoing treatment (n=5), no unmet needs (n=9)	95.7% follow-up rate at T1 and 94.7% follow-up rate at T2. Intervention group had a 95.7% adherence rate.	Did not return questionnaire (n=2 at T1; n=3 at T2)	NR	NR
Korstjens 2008, Korstjens 2011, May 2008, May 2009, van Weert 2010	83.5% (147/176) randomised/self-referred	Not eligible (n=14), refused to participate (n=5), refused randomisation assignment (n=7), felt no need for rehabilitation anymore (n=3)	Adherence: 83.5% of 24 physical training sessions completed by each group. PT+CBT group completed 82.4% of 12 CBT sessions.	Withdrawal: 8% (6 of 76) discontinued PT+CBT, 13% (9 of 71) discontinued PT. Medical reasons (n=11) Personal reasons (n=4)	NR	NR
Krouse 2016, Hornbrook 2018, Cidav 2021	NR[a]	NR	9 (of 38) participants withdrew (1 before Session 1, 3 before Session 2, and 5 before Session 4).	No longer wanting to participate (n=3), death (n=2), illness (n=2), early	Sessions were initially approximately every 3 weeks, but midway through the program,	NR

Study	Recruitment rate	Reasons for non-participation	Intervention adherence	Reasons for withdrawal	Intervention modifications	Fidelity to the protocol
			Among those who had not dropped out attendance was relatively high (Session 1 = 100%, Session 2 = 97.1%, Session 3 was for caregivers only, Session 4 = 93.1%, and Session 5 which included caregivers also = 96.6%). Outcome measures available for 25 pre-session, 23 post-session, and 23 follow-up, of which 21 completed all three surveys.	ostomy reversal (n=1), travel distance (n=1).	based on participant comments, was changed to Sessions 1 and 2 being on one day, followed by sessions 3, 4 and 5 approximately one month later. Inclusion criteria widened during the study to include all survivors with ostomies.	
Kvale 2016	2.2% (79/3640) randomised/assessed for eligibility	Not eligible (n=3446); declined (n=15); other barriers (n=99); no show after consent (n=1)	Lost to follow up (n=2)	Withdrawal due to cancer recurrence (n=1); could not be reached (n=1)	NR	Achieved fidelity at a level of 75%
Lawn 2015, Miller 2016	34.7% (25/72) eligible and agreed to participate/approached	The main reason for declining was lifestyle changes not being considered a priority	22 participants (91%) “completed the FLW Program and other assessments at 6 weeks” but only 20 (80%) completed them at 12 weeks.	Coping issues (n=2), cancer recurrence (n=1), chemotherapy-related complications (n=1)	NR	NR
Lee 2010	13.4% (33/246) accepted/invited based on inclusion criteria	NR	12 withdrawals (36%). 50% withdrawn within three months.	Returned to work (n=4), lack of time (n=2), child care responsibilities (n=1), hospitalized (n=1), died (n=1), reason not stated (n=3).	NR	NR
Lee 2014	17.2% (59/343) randomised/assessed for eligibility	Excluded for medical reasons (n=112), not contactable/withdrew interest (n=59), already practicing exercise and dietary behaviours (n=57), did not participate in screening tests (n=34), no internet access	1 participant withdrew	Breast cancer recurrence (n=1)	NR	NR

Study	Recruitment rate	Reasons for non-participation	Intervention adherence	Reasons for withdrawal	Intervention modifications	Fidelity to the protocol
		(n=8), unfamiliar with the internet (n=14)				
Loubani 2021	43.2% (35/81) randomised/assessed for eligibility	Not meeting inclusion criteria (n=21), declined to participate (n=17), other reasons (n=8)	Lost during intervention (n=1), lost at T2 (n=1), lost at T3 (n=1)	Non-compliance (n=1), other reasons not reported	NR	NR
Manne 2020	15.9% (66/415) participated/assessed for eligibility	Refused participation (n=246), ineligible (n=95), could not be located (n=3), did not complete baseline (n=5)	86.4% completion rate at T1, 89.4% completion rate at T2. 90.9% participants logged in, 81.8% viewed 3/4 of the 4 modules. Time spent in intervention ranged from 0 to 272 minutes (average 85 minutes)	NR	NR	NR
Mardani 2020	37.9% (80/211) allocated/assessed for eligibility	Not meeting inclusion criteria (n=85), declined to participate (n=21)	Lost to follow up (n=5), 80.1% adherence rate in the intervention group.	Discontinued intervention (n=3), no willingness (n=2)	NR	NR
McCusker 2021	28.2% (245/870) randomised/referred to study	Not meeting inclusion criteria (n=361), could not contact (n=100), not interested (n=103), consent form not returned (n=52), suicidal intent (n=4), no longer interested (n=2), did not complete baseline (n=3)	82.6% of intervention group completed follow-up.	Incomplete primary outcome (n=2), drop out (n=8), suicidal intent (n=1), lost to follow-up (n=10)	NR	NR
Meneses 2017	2.7% (40/1473) enrolled/assessed for eligibility	Declined (n=131), mail-shot was returned (n=220), no response (1029), did not enrol (n=53 reasons not given)	NR	NR	NR	NR
Moon 2019	15.5% (41/264) consented/assessed for eligibility	Not eligible (n=171), not interested (n=1), too busy (n=2), reason not given (n=3)	8 (22%) did not complete baseline questionnaire. 5 (12%) did not complete intervention. 28 (68%) of recruited sample reported to have completed intervention materials, however, the authors report that some participants only engaged with	Reason not given (n=8), stressful life events (n=2), too busy (n=2), no longer wants to participate (n=1)	NR	NR

Study	Recruitment rate	Reasons for non-participation	Intervention adherence	Reasons for withdrawal	Intervention modifications	Fidelity to the protocol
			certain sections of written material or did not complete the activities.			
Newman 2019	10.3% (15/146) enrolled/assessed for eligibility	Not eligible (n=116), Unable to contact (n=3), not referred by provider (n=8), not interested in group program (n=2), conflict with work schedule (n=1), too far to travel (n=1)	73% attended at least five of six sessions.<br>1 withdrawal prior to first session.	Inability to attend the scheduled sessions, moving out of the area, difficulties contacting participants for follow-up assessments.	NR	NR
Omidi 2020	100% (105/105) randomised/assessed for eligibility	NA	Excluded from follow-up (n=4)	Failure to receive messages during the intervention (n=1), absence in third and fourth sessions (n=3)	NR	NR
Salvatore 2015, Ahn 2013, Ory 2013	NR	NR	NR	NR	NR	NR
Schmidt 2016	84% (79/94) enrolled/eligible	NR	Lost to follow up at 60 days following transplantation (n=5); lost to follow up at 100 days (n=8)	NR	NR	Organisational barriers to delivery of the sports therapy module and inability to deliver the nutritional module to the planned intensity due to lack of patient need.
Skolarus 2019	83.5% (556/666) randomised/assessed for eligibility	Not eligible (n=38), declined to participate (n=61), contact limit reached before random assignment (n=11)	Completed 5-month staff survey (n=244/278), completed 5-month EPIC (n=242/278)	Deceased (n=1), withdrew consent (n=16), withdrawn by study staff (n=1), lost to follow-up (n=16)	NR	NR
Turner 2019	NR	NR	3-month follow-up (n=5); 6-month follow-up (n=5);<br>1 of 14 participants interviewed afterwards had used the plan, although they all remembered the interview. None discussed their plan with their doctor as	Unable to contact (n=7), medical decline (n=2), death (n=1)	NR	NR

Study	Recruitment rate	Reasons for non-participation	Intervention adherence	Reasons for withdrawal	Intervention modifications	Fidelity to the protocol
			instructed. Two used the 'Facing the Future' information resource.			
Van den Berg 2015, Van den Berg 2013	88% (150/170) randomised/referred as eligible	Intervention did not meet needs (n=6), time investment (n=5), assessments too confronting (n=4), web-based nature (n=2) participating in other trials (n=1), unknown (n=1), metastases (n=1)	Frequency of log ins ranged from 0-45; duration ranged from 0-2324 minutes; activity ranged from 0-104 (of 104) intervention components opened	NR	NR	NR
Van der Hout 2020a, Van der Hout 2020b, Van der Hout 2021a, Van der Hout 2021b, Duman-Lubberding 2016	21.2% (625/2953) randomised/assessed for eligibility	No response (n=1874), not interested (n=243), no internet and/or email (n=211)	19% (60/320) participants withdrew by 6-month follow-up	Questions too burdensome or time consuming (n=23), poor health (n=10), lacking internet skills (n=7), personal circumstances (n=9), questions not applicable (n=9), unknown (n=2)	NR	52% used Oncokompas as intended.
Watson 2018, Burns 2017	15.2% (83/546) allocated/assessed for eligibility	Declined to participate (n=177), not eligible (n=53)	5% (2/42) patients discontinued the intervention. Lost at 6-month follow-up (n=1)	Disease progression (n=1), unrelated health reasons (n=1)	NR	Regular meetings between nurses delivering intervention and study lead. Some nurses felt de-skilled in the long gaps between appointments.
Willems 2016, Willems 2017a, Willems 2017b, Kanera 2016a, Kanera 2016b, Kanera 2017	39.8% (518/1303) randomised/assessed for eligibility	Declined (n=100), computer literacy (n=10), not meeting inclusion criteria (n=5), unknown (n=670)	At 6 months, 85.7% had accessed at least one module. Participants used on average 2.1 (SD 1.6) of eight modules. 14.3% used no modules, 30.3% used one, 18.2% used two, 21.2% used three 8.7% used four, 3.9% used five, 3.4% used six or more.	Did not give consent (n=6), deceased (n=4), not meeting inclusion criteria (n=15), other (n=39)	NR	NR
Yun 2012	24.7% (273/1106) randomised/assessed for eligibility	Declined participation (n=796), ineligible (n=37),	83% (113/136) completed the 12-week course.	Too busy (n=7), withdrew (n=11), poor internet use (n=5)	NR	NR

Study	Recruitment rate	Reasons for non-participation	Intervention adherence	Reasons for withdrawal	Intervention modifications	Fidelity to the protocol
		screen failure (n=18), questionnaire refused (n=19)				
Zhang 2015	21% (279/1331) randomised/assessed for eligibility	Not eligible (n=595), refused (n=253), out of reach (n=144), withdrew prior to randomisation (n=10)	13% (35/279) participants withdrew	Eligibility change, worsening health, family or economic conditions	NR	The Problem Solving Therapy element of the intervention was manualised. Authors report that 'reliability across therapists was examined to ensure consistency and adherence'

[a]There was no clear denominator to determine recruitment rate.
NR = not reported

Table S8: Critical appraisal skills programme (CASP) risk of bias appraisal

Study	Clearly focused issue?	Randomised assignment to groups?	All patients who entered trial accounted for at conclusion?	Patients, health workers and study personnel blind to treatment?	Groups similar at start of trial?	Groups treated equally outwith intervention?
Chambers 2018	Yes	Yes	Yes	Study personnel yes, Patients no	Can't tell	Can't tell
Foster 2016	Yes	Yes	Yes	Study personnel yes, Patients no	Yes	Yes
Gregoire 2020, Gregoire 2021	Yes	Yes	Yes	Patients yes, Study personnel no	Yes	Yes
Kim 2021	Yes	Yes	Yes	No	Yes	Yes
Korstjens 2008, Korstjens 2011, May 2008, May 2009, van Weert 2010	Yes	Yes	Yes	No	Yes	Can't tell
Kvale 2016	Yes	Yes	Yes	No	Yes	Yes
Lee 2014	Yes	Yes	Yes	No	Yes	Can't tell
Loubani 2021	Yes	Yes	Yes	Study personnel yes, Patients no	Yes	Yes
Mardani 2020	Yes	Yes	Yes	Study personnel yes, Patients no	Yes	Yes
McCusker 2021	Yes	Yes	Yes	Study personnel yes, Patients no	Yes	Yes
Meneses 2017	Yes	Yes	No	No	Can't tell	Can't tell
Omidi 2020	Yes	Yes	Yes	Study personnel yes, Patients no	Yes	Yes
Skolarus 2019	Yes	Yes	Yes	No	Yes	Yes
Turner 2019	Yes	Yes	Yes	No	Yes	Yes
Van den Berg 2015, Van den Berg 2013	Yes	Yes	No	No	Yes	Yes

Study	Clearly focused issue?	Randomised assignment to groups?	All patients who entered trial accounted for at conclusion?	Patients, health workers and study personnel blind to treatment?	Groups similar at start of trial?	Groups treated equally outwith intervention?
Van der Hout 2020a, Van der Hout 2020b, Van der Hout 2021a, Van der Hout 2021b, Duman-Lubberding 2016	Yes	Yes	Yes	No	Yes	Can't tell
Watson 2018, Burns 2017	Yes	Yes	No	No	Yes	Can't tell
Willems 2016, Willems 2017a, Willems 2017b, Kanera 2016a, Kanera 2016b, Kanera 2017	Yes	Yes	No	No	Yes	Can't tell
Yun 2012	Yes	Yes	No	No	Yes	Yes
Zhang 2015	Yes	Yes	Yes	Yes	Yes	Yes

Table S9: Joanna Briggs Institute (JBI) risk of bias appraisal

Study	Clear cause and effect?	Similar participants in comparisons?	Comparison participants receiving similar treatment outwith intervention?	Control group?	Multiple measurements of outcome both pre and post?	Follow-up complete? If not, group differences adequately analysed?	Participant outcomes measured in same way?	Outcomes measured in a reliable way?	Appropriate statistical analysis?
Faithfull 2010	Yes	Yes	NA	No	No	Yes	Yes	Unclear	Unclear
Frankland 2019	Yes	Yes	Yes	Yes	No	Yes	Yes	Unclear	Yes
Fu 2016	Yes	Yes	NA	No	No	Unclear	Yes	Unclear	Yes
Kazer 2011	Yes	Yes	NA	No	No	No	Yes	Yes	Unclear
Krouse 2016, Hornbrook 2018, Cidav 2021	Yes	Yes	NA	No	No	Yes	Yes	Unclear	Unclear
Lawn 2015, Miller 2016	Yes	Yes	NA	No	No	Yes	Yes	Unclear	Unclear
Lee 2010	Yes	Yes	NA	No	No	Yes	Yes	Yes	Unclear
Manne 2020	Yes	Yes	NA	No	No	Unclear	Yes	Yes	Yes
Moon 2019	Yes	Yes	NA	No	No	Yes	Yes	Unclear	Unclear
Newman 2019	Yes	Yes	NA	No	No	Yes	Yes	Yes	Unclear
Salvatore 2015, Ahn 2013, Ory 2013	Yes	Yes	NA	No	No	No	Yes	Unclear	Yes
Schmidt 2016	Yes	Yes	Yes	Yes	No	No	Yes	Unclear	Yes

Table S10: Quality of life instruments and their scoring

Instrument	Citation	Dimensions assessed	Scoring
AQoL-8D	Richardson, J., Iezzi, A., Khan, M. A., & Maxwell, A. (2014). Validity and reliability of the Assessment of Quality of Life (AQoL)-8D multi-attribute utility instrument. The Patient-Patient-Centered Outcomes Research, 7(1), 85-96.	Independent living, pain, senses, mental health, happiness, coping, relationships, self-worth	Subscale and total scores. Higher score indicates better QoL.
BCLE-SEI	Fu, M. R., Cleland, C. M., & Kang, Y. (2012, June). Measuring lymphedema symptom burdens: a psychometric study. In Multinational Association of Supportive Care in Cancer's Annual Meeting (MASCC/ISOO).	Pain, aching, soreness, tenderness, lymphedema symptoms	Individual item scores. Higher score indicates worse QoL. Categorical 'yes' or 'no' or five-point scale from 0 (no presence of a given symptom) to 4 (greatest severity of a given symptom).
CoH-QoL-O	Grant, M., Ferrell, B., Dean, G., Uman, G., Chu, D., & Krouse, R. (2004). Revision and psychometric testing of the City of Hope Quality of Life–Ostomy Questionnaire. Quality of Life Research, 13(8), 1445-1457.	Physical, psychological, social, spiritual wellbeing	Subscale and total scores. Higher score indicates better QoL. 11 point scale from 0 to 10 (anchors not given).
EORTC QLQ-BR23	Sprangers, M. A., Groenvold, M., Arraras, J. I., Franklin, J., te Velde, A., Muller, M., ... & Aaronson, N. K. (1996). The European Organization for Research and Treatment of Cancer breast cancer-specific quality-of-life questionnaire module: first results from a three-country field study. Journal of clinical oncology, 14(10), 2756-2768.	Body image, sexual functioning, sexual enjoyment, future perspective, systemic therapy side effects, breast symptoms, arm symptoms, upset by hair loss	Subscale and total scores. Higher functioning score indicates better QoL. Higher symptom score indicates worse QoL. Four-point scale from 1 (Not at all) to 4 (Very much) for how much experienced an issue in the last week.
EORTC QLQ-C30	Aaronson, N. K., Ahmedzai, S., Bergman, B., Bullinger, M., Cull, A., Duez, N. J., ... & Takeda, F. (1993). The European Organization for Research and Treatment of Cancer QLQ-C30: a quality-of-life instrument for use in international clinical trials in oncology. JNCI: Journal of the National Cancer Institute, 85(5), 365-376.	Physical, role, emotional, cognitive, social functioning, fatigue, nausea, pain, dyspnoea, insomnia, appetite loss, constipation, diarrhoea, financial difficulties	Subscale and total scores. Higher functioning score indicates better QoL. Higher symptom score indicates worse QoL. Four-point scale from 1 (Not at all) to 4 (Very much) for how much experienced an issue in the last week.
EORTC QLQ-CR29	Whistance, R. N., Conroy, T., Chie, W., Costantini, A., Sezer, O., Koller, M., ... & Blazeby, J. M. (2009). Clinical and psychometric validation of the EORTC QLQ-CR29 questionnaire module to assess health-related quality of life in patients with colorectal cancer. European journal of cancer, 45(17), 3017-3026.	Urinary frequency, blood and mucus in stool, stool frequency, body image, urinary incontinence, dysuria, abdominal pain, buttock pain, bloating, dry mouth, hair loss, taste, anxiety, weight, flatulence, faecal incontinence, sore skin, embarrassment, stoma care problems, sexual interest (men), impotence (men), sexual interest (women), dyspareunia (women)	Subscale and total scores. Higher functioning score indicates better QoL. Higher symptom score indicates worse QoL. Four-point scale from 1 (Not at all) to 4 (Very much) for how much experienced an issue in the last week.
EORTC QLQ-HN35	Bjordal, K., Hammerlid, E., Ahlner-Elmqvist, M., De Graeff, A., Boysen, M., Evensen, J. F., ... & Kaasa, S. (1999).	Pain, swallowing, senses, speech, social eating, social contact, sexuality, problems	Subscale and total scores. Higher functioning score indicates better QoL. Higher symptom score

Instrument	Citation	Dimensions assessed	Scoring
	Quality of life in head and neck cancer patients: validation of the European Organization for Research and Treatment of Cancer Quality of Life Questionnaire-H&N35. Journal of Clinical Oncology, 17(3), 1008-1008.	with teeth, problems opening mouth, dry mouth, sticky saliva, coughed, felt ill, painkillers, nutritional supplements, feeding tube, lost weight, gained weight	indicates worse QoL. Four-point scale from 1 (Not at all) to 4 (Very much) for how much experienced an issue in the last week.
EORTC QLQ-HN43	Singer, S., Amdal, C. D., Hammerlid, E., Tomaszewska, I. M., Castro Silva, J., Mehanna, H., ... & EORTC Quality of Life and the EORTC Head and Neck Cancer Groups. (2019). International validation of the revised European Organisation for Research and Treatment of Cancer Head and Neck Cancer Module, the EORTC QLQ-HN43: Phase IV. Head & neck, 41(6), 1725-1737.	Fear of progression, body image, dry mouth and sticky saliva, pain in the mouth, sexuality, problems with senses, problems with shoulder, skin problems, social eating, speech, swallowing, problems with teeth, coughing, swelling in the neck, neurological problems, trismus, social contact, weight loss, problems with wound healing	Subscale and total scores. Higher functioning score indicates better QoL. Higher symptom score indicates worse QoL. Four-point scale from 1 (Not at all) to 4 (Very much) for how much experienced an issue in the last week.
EORTC QLQ-NHL-HG29	van de Poll-Franse, L., Oerlemans, S., Bredart, A., Kyriakou, C., Sztankay, M., Pallua, S., ... & Efficace, F. (2018). International development of four EORTC disease-specific quality of life questionnaires for patients with Hodgkin lymphoma, high- and low-grade non-Hodgkin lymphoma and chronic lymphocytic leukaemia. Quality of life research, 27(2), 333-345.	Symptom burden, neuropathy, physical condition/fatigue, emotional impacts, worries/fears about health and functioning	Subscale and total scores. Higher functioning score indicates better QoL. Higher symptom score indicates worse QoL. Four-point scale from 1 (Not at all) to 4 (Very much) for how much experienced an issue in the last week.
EORTC QLQ-PR25	Van Andel, G., Bottomley, A., Fosså, S. D., Efficace, F., Coens, C., Guerif, S., ... & Aaronson, N. K. (2008). An international field study of the EORTC QLQ-PR25: a questionnaire for assessing the health-related quality of life of patients with prostate cancer. European journal of cancer, 44(16), 2418-2424.	Urinary symptoms, bother due to the use of incontinence aid, bowel symptoms, hormonal treatment-related symptoms, sexual activity, sexual functioning	Subscale and total scores. Higher functioning score indicates better QoL. Higher symptom score indicates worse QoL. Four-point scale from 1 (Not at all) to 4 (Very much) for how much experienced an issue in the last week.
EPIC-26	Szymanski, K. M., Wei, J. T., Dunn, R. L., & Sanda, M. G. (2010). Development and validation of an abbreviated version of the expanded prostate cancer index composite instrument for measuring health-related quality of life among prostate cancer survivors. Urology, 76(5), 1245-1250.	Urinary incontinence, urinary irritation, bowel function, sexual function, hormonal function	Subscale scores. 0-100 scores with higher score indicates better QoL. Variety of scales and anchors used.
FACT-B	Brady, M. J., Cella, D. F., Mo, F., Bonomi, A. E., Tulsky, D. S., Lloyd, S. R., ... & Shiomoto, G. (1997). Reliability and validity of the Functional Assessment of Cancer Therapy-Breast quality-of-life instrument. Journal of clinical oncology, 15(3), 974-986.	Physical well-being, social/family well-being, emotional well-being, functional well-being, breast cancer subscale	Subscale and total scores. Higher score indicates better QoL. Five-point scale from 0 (Not at all) to 4 (Very much) for how much experienced an issue in the past week.
FACT-Cog	Wagner, L. I., Lai, J. S., Cella, D., Sweet, J., & Forrestal, S. (2004). Chemotherapy-	Perceived cognitive impairments, impact of	Subscale and total scores. Higher score indicates better QoL. Five-

Instrument	Citation	Dimensions assessed	Scoring
	related cognitive deficits: development of the FACT-Cog instrument. Ann Behav Med, 27(Suppl 10).	perceived cognitive impairments on QoL, comments from others, perceived cognitive abilities	point scale from 0 (Never) to 4 (Several times a day) for how much experienced an issue in the past week.
FACT-ES	Fallowfield, L. J., Leaity, S. K., Howell, A., Benson, S., & Cella, D. (1999). Assessment of quality of life in women undergoing hormonal therapy for breast cancer: validation of an endocrine symptom subscale for the FACT-B. Breast cancer research and treatment, 55(2), 187-197.	Physical well-being, social/family well-being, emotional well-being, functional well-being, endocrine symptom subscale	Subscale and total scores. Higher score indicates better QoL. Five-point scale from 0 (Not at all) to 4 (Very much) for how much experienced an issue in the past week.
FACT-G	Cella, D. F., Tulsky, D. S., Gray, G., Sarafian, B., Linn, E., Bonomi, A., ... & Bonomi, P. (1993). The Functional Assessment of Cancer Therapy scale: development and validation of the general measure. J Clin Oncol, 11(3), 570-579.	Physical well-being, social/family well-being, emotional well-being, functional well-being	Subscale and total scores. Higher score indicates better QoL. Five-point scale from 0 (Not at all) to 4 (Very much) for how much experienced an issue in the past week.
FACT-H&N	List, M. A., D'Antonio, L. L., Cella, D. F., Siston, A., Mumby, P., Haraf, D., & Vokes, E. (1996). The performance status scale for head and neck cancer patients and the functional assessment of cancer therapy-head and neck scale: a study of utility and validity. Cancer: Interdisciplinary International Journal of the American Cancer Society, 77(11), 2294-2301.	Physical well-being, social/family well-being, emotional well-being, functional well-being, head and neck cancer subscale	Subscale and total scores. Higher score indicates better QoL. Five-point scale from 0 (Not at all) to 4 (Very much) for how much experienced an issue in the past week.
LLIS	Weiss, J., & Daniel, T. (2015). Validation of the lymphedema life impact scale (LLIS): a condition-specific measurement tool for persons with lymphedema. Lymphology, 48(3), 128-138.	Physical concerns, psychosocial concerns, functional concerns	Subscale and total scores. Higher score indicates worse QoL. Five-point scale from 1 (no impairment) to 5 (severe impairment).
PCI	Litwin, M. S., Hays, R. D., Fink, A., Ganz, P. A., Leake, B., & Brook, R. H. (1998). The UCLA Prostate Cancer Index: development, reliability, and validity of a health-related quality of life measure. Medical care, 1002-1012.	Physical functioning, role functioning - physical, bodily pain, general health, vitality, social functioning, role functioning - emotional, mental health, urinary function, sexual function, bowel function, urinary bother, sexual bother, bowel bother	Subscale scores. Higher score indicates better QoL. Variety of scales and anchors used.
SF-12	Ware Jr, J. E., Kosinski, M., & Keller, S. D. (1996). A 12-Item Short-Form Health Survey: construction of scales and preliminary tests of reliability and validity. Medical care, 220-233.	Physical functioning, role functioning - physical, bodily pain, general health, vitality, social functioning, role functioning - emotional, mental health, physical component scores, mental component scores	Subscale scores. Higher score indicates better QoL. Variety of scales and anchors used.

Instrument	Citation	Dimensions assessed	Scoring
SF-36	Brazier, J. E., Harper, R., Jones, N. M., O'cathain, A., Thomas, K. J., Usherwood, T., & Westlake, L. (1992). Validating the SF-36 health survey questionnaire: new outcome measure for primary care. British medical journal, 305(6846), 160-164.	Physical functioning, role functioning - physical, bodily pain, general health, vitality, social functioning, role functioning - emotional, mental health, physical component scores, mental component scores	Subscale scores. Higher score indicates better QoL. Variety of scales and anchors used.

AQoL-8D = Assessment of Quality of Life – 8 dimensions; BCLE-SEI = Breast Cancer and Lymphedema Symptom Experience Index; CoH-QoL-O = City of Hope Quality of Life Ostomy questionnaire; EORTC QLQ-BR23 = European Organisation for Research and Treatment of Cancer Quality of life Questionnaire Breast; EORTC QLQ-C30 = European Organisation for Research and Treatment of Cancer Quality of life Questionnaire Core; EORTC QLQ-CR29 = European Organisation for Research and Treatment of Cancer Quality of life Questionnaire Colorectal; EORTC QLQ-HN35 = European Organisation for Research and Treatment of Cancer Quality of life Questionnaire Head and Neck; EORTC QLQ-HN43 = European Organisation for Research and Treatment of Cancer Quality of life Questionnaire Head and Neck; EORTC QLQ-NHL-HG29 = European Organisation for Research and Treatment of Cancer Quality of life Questionnaire Low Grade Non-Hodgkin's Lymphoma; EORTC QLQ-PR25 = European Organisation for Research and Treatment of Cancer Quality of life Questionnaire Prostate; EPIC-26 = Expanded Prostate Cancer Index Composite; FACT-B = Functional Assessment of Cancer Therapy - Breast; FACT-Cog = Functional Assessment of Cancer Therapy – Cognitive function; FACT-ES = Functional Assessment of Cancer Therapy – Endocrine Symptoms; FACT-G = Functional Assessment of Cancer Therapy – General; FACT-H&N = Functional Assessment of Cancer Therapy – Head & Neck; LLIS = Lymphedema Life Impact Scale; PCI = Prostate Cancer Index; QoL = Quality of Life; SF-12 = 12-Item Short Form Survey; SF-36 = Short Form 36 health survey questionnaire

Table S11: Self-efficacy and additional outcomes

Study	Was self-efficacy an outcome?	Instrument(s) used	Timepoint(s) measured	Any significant differences reported?	Other outcomes reported
Chambers 2018	No	NA	NA	NA	Post-traumatic growth, Unmet needs
Faithfull 2010	Yes	Stanford Patient Education Research Centre 6-item scale	Baseline, 6 months	No	NR
Foster 2016	Yes	PSEFSM, CS-SES	Baseline, 6 weeks, 12 weeks	No	Depression, Fatigue, Personal wellbeing
Frankland 2019	No	NA	NA	NA	Fear of cancer recurrence, General health behaviours, Patient activation, Psychological wellbeing, Satisfaction with care, Treatment side effects, Unmet needs
Fu 2016	No	NA	NA	NA	Self-care behaviour
Gregoire 2020, Gregoire 2021	No	NA	NA	NA	Emotional distress, Emotional regulation, Fatigue, Mindfulness, Physical activity, Self-esteem, Sleep difficulties
Kazer 2011	Yes	Adaptation of Lorig	Baseline, 5 weeks, 10 weeks	No	Illness uncertainty
Kim 2021	Yes	CS-SES	Baseline, 8 weeks, 20 weeks	From baseline to 8 weeks, self-efficacy for self-management significantly improved in the intervention group (p=.026).	Anxiety, Depression, Empowerment, Psychosocial adjustment

Study	Was self-efficacy an outcome?	Instrument(s) used	Timepoint(s) measured	Any significant differences reported?	Other outcomes reported
Korstjens 2008, Korstjens 2011, May 2008, May 2009, van Weert 2010	No	NA	NA	NA	Anxiety, Depression, Physical activity, Problem solving
Krouse 2016, Hornbrook 2018, Cidav 2021	Yes	Adaptation of Lorig	Baseline, post-intervention, 6 months	Self-efficacy for ostomy management significantly improved from baseline to follow-up (p=.008).	Anxiety, Depression, Patient activation, Satisfaction with Ostomy Care
Kvale 2016	Yes	Self-efficacy for Managing Chronic Disease 6-item scale	Baseline, 3 months	No	Depression, Self-management, Social/role activities
Lawn 2015, Miller 2016	No	NA	NA	NA	Anthropometry, Body composition, Diet, Functional exercise capacity, Physical activity, Strength, Self-management
Lee 2010	No	NA	NA	NA	Depression, Immune markers
Lee 2014	Yes	Author designed questions, perceived self-efficacy for exercise, perceived self-efficacy to eat at least 5 servings of fruit & veg a day	Baseline, 12 weeks	At 12 weeks follow-up the intervention group had significantly greater self-efficacy for exercise management (p=.024) and to increase fruit and vegetable intake (p=.023) than the control group.	Anxiety, Depression, Diet, Exercise, Fatigue, Fruit & veg servings, Stage of change
Loubani 2021	No	NA	NA	NA	Activity level, Cognitive function,

Study	Was self-efficacy an outcome?	Instrument(s) used	Timepoint(s) measured	Any significant differences reported?	Other outcomes reported
					Executive function, Hand-grip strength, Occupational performance, Upper extremity disability
Manne 2020	Yes	Author designed scale to manage oral and oropharyngeal cancer care	Baseline, 2 months, 6 months	Self-efficacy significantly improved from baseline to 2 months (p<.01). This improvement was maintained at 6 months.	Action and coping planning, Information needs, Patient activation, Performance and thoroughness of oral self-exams, Performance of maintenance exercises, Preparedness, Support needs
Mardani 2020	No	NA	NA	NA	NR
McCusker 2021	No	NA	NA	NA	Anxiety, Depression, Patient activation
Meneses 2017	No	NA	NA	NA	NR
Moon 2019	Yes	Adaptation of Lorig	Baseline, post-intervention	From pre- to post-intervention self-efficacy significantly improved for managing hot flushes in those with moderate/severe hot flushes (p=.027); managing night sweats in those with moderate/severe night sweats (p=.043); managing leg cramps/joint pain in those with leg cramps/joint pain (p=.039) and those with moderate/severe leg cramps/joint pain (p=.007); managing vaginal health problems in those with vaginal health problems (p=.002) and those with moderate/severe vaginal health problems (p=.005); managing mood changes in those with moderate/severe symptoms (p=.008); and managing fatigue (p=.001).	Distress, Illness and treatment beliefs
Newman 2019	No	NA	NA	NA	Activity level, Occupational performance

Study	Was self-efficacy an outcome?	Instrument(s) used	Timepoint(s) measured	Any significant differences reported?	Other outcomes reported
Omidi 2020	No	NA	NA	NA	Fear of cancer recurrence
Salvatore 2015, Ahn 2013, Ory 2013	No	NA	NA	NA	Communication with physicians, Depression, Fatigue, Health status, Medication compliance, Pain, Role function, Shortness of breath, Sleep difficulties, Stress
Schmidt 2016	Yes	G-SES	Day before HSCT, 60th day following transplantation	No	Blood parameters, Body mass index, Compliance, Physical activity
Skolarus 2019	No	NA	NA	NA	Cancer control, Cancer outlook, Communication with physicians, Coping
Turner 2019	Yes	CBI	Baseline, 3 months, 6 months	Total self-efficacy significantly improved in the intervention group from baseline to 3 months (p<.05) and 6 months (p<.01) and in the information group from baseline to 6 months (p<.01). Self-efficacy for maintenance of activity and independence significantly improved from baseline to 3 and 6 months in the intervention (p<.05) and information (p<.01) groups, and from baseline to 6 months in the usual care group (p<.05). Self-efficacy for stress management and coping with treatment related side effects significantly improved from baseline to 6 months in the information group (p<.05). Self-efficacy for accepting cancer/maintaining a positive attitude significantly improved from baseline to	Anxiety, Depression

Study	Was self-efficacy an outcome?	Instrument(s) used	Timepoint(s) measured	Any significant differences reported?	Other outcomes reported
				6 months in the intervention group (p<.05). Self-efficacy for affective regulation significantly improved from baseline to 3 months in the intervention group (p<.05), and from baseline to 6 months in the information group (p<.05). Self-efficacy for seeking social support significantly improved from baseline to 6 months in the intervention and information groups (p<.05).	
Van den Berg 2015, Van den Berg 2013	Yes	G-SES	Baseline, 4 months, 6 months, 10 months	At the 4 month follow-up point, self-efficacy was significantly greater in the intervention group, than the control group (p<.05).	Acceptance,<br>Distress,<br>Empowerment,<br>Fatigue,<br>Fear of recurrence,<br>Fulfilment,<br>Helplessness,<br>New ways of living,<br>Perceived benefits,<br>Personal control,<br>Re-evaluation,<br>Remoralisation,<br>Valuing life
Van der Hout 2020a, Van der Hout 2020b, Van der Hout 2021a, Van der Hout 2021b, Duman-Lubberding 2016	Yes	G-SES	Baseline, 1 week, 3 months, 6 months	No	Communication with physicians,<br>Mental adjustment to cancer,<br>Patient activation,<br>Personal control,<br>Unmet needs
Watson 2018, Burns 2017	Yes	CS-SES	Baseline, 7 months	No	Anxiety,<br>Depression,<br>Unmet needs

Study	Was self-efficacy an outcome?	Instrument(s) used	Timepoint(s) measured	Any significant differences reported?	Other outcomes reported
Willems 2016, Willems 2017a, Willems 2017b, Kanera 2016a, Kanera 2016b, Kanera 2017	No	NA	NA	NA	Anxiety, Depression, Diet, Fatigue, Personal control, Physical activity, Problem solving
Yun 2012	No	NA	NA	NA	Distress, Energy conservation, Fatigue, Nutrition, Pain, Physical activity, Sleep difficulties
Zhang 2015	No	NA	NA	NA	NR

CBI = Cancer behaviour inventory; CS-SES = Cancer survivors' self-efficacy scale; G-SES = General self-efficacy scale; HSCT = Hematopoietic stem cell transplantation; NA = not applicable; NR = not reported; PSEFSM = Perceived self-efficacy for fatigue self-management

Table S12: Economics

Study	Outcome(s) assessed	Instrument(s) used	Timepoint(s) measured	Outcomes
**Foster 2016**	Health service resource use	Number of visits to GP and oncologist	12 weeks	At 12 weeks since baseline, intervention group (2.29, SD 1.27; 1.41, SD 0.80) had slightly more visits to GP and oncologist than control group (1.90, SD 1.04; 1.29, SD 1.27). This was not statistically significant.
**Frankland 2019**	Health service resource use, Patient costs	15 questions about contacts and costs, related to prostate cancer	4 and 8 months	Direct cost of provision of the intervention was £102 (GBP 2015) per patient, compared to £59 for the comparators. Combining direct costs and costs of service use, the programme group had lower overall average costs of £289 per patient compared to £327 for the comparator group.
Krouse 2016, **Hornbrook 2018, Cidav 2021**	Cost of provision of intervention, from payer perspective	Time-driven activity-based costing	Across the 2 year study period	Per-patient cost of OSMT was $1758 (USD 2020), with personnel time accounting for 91% of the cost. Intervention coordination and monitoring efforts were 62% of total time cost. The total incremental expense per intervention cohort of 4 survivors was $7246 or $1812 per patient.
**McCusker 2021**	Health service resource use	Number of visits to: emergency room, GP, oncologist, mental health specialist, other specialist, complementary medicine specialist (e.g. acupuncture)	Baseline, 6 months	Adjusted incidence rate ratio (IRR, with 95% CI) for visits across the 6 month period, intervention vs control groups: emergency room (0.47, 0.21-1.08); GP (0.89, 0.61-1.30); oncologist (1.27, 0.80-2.00); mental health (1.12, 0.34-3.63); other specialist (1.24, 0.79-1.94); and complementary medicine (0.52, 0.14-1.86). None of these were statistically significant.
Salvatore 2015, **Ahn 2013**, Ory 2013[b]	Health service resource use, Healthcare cost savings	Self-reported healthcare utilisation among those using the intervention; comparator data from 2010 Medical expenditure panel survey	Baseline, 6 months, 12 months	Significant reductions in emergency room visits (5%) at 6- and 12-month assessments, and hospitalisations (3%) at 6-months among intervention participants. Equates to potential net savings of $364 per participant and national saving of $3.3 billion if 5% of adults with one or more chronic conditions were reached.
**Schmidt 2016**	Health service resource use	Duration of hospitalisation and use of antibiotics	Day before transplant, day before discharge	Intervention group (mean 2.0, SE 0.9) prescribed more antibiotics at t2 than control group (mean 1.1, SE 0.2; mean difference 0.9, 95% CI 0.5-1.3); Duration of hospitalisation was mean 10.9 days (95% CI -18.0 to -3.7) shorter in intervention group (23.5, SE 3.6) than control group (34.4, SE 3.9)
**Skolarus 2019**	Cost of provision of intervention, from payer perspective	Cost of automated call minutes	Across the one year study period	Total estimated call costs for the study were $531, and the average control and intervention participant call costs were $0.65 and $1.40, respectively.

Study	Outcome(s) assessed	Instrument(s) used	Timepoint(s) measured	Outcomes
Van der Hout 2020a, **Van der Hout 2020b**, Van der Hout 2021a, Van der Hout 2021b, Duman-Lubberding 2016	Cost-utility analysis from societal perspective, Direct (non-) medical costs, indirect (non-) medical costs, QALYs	iMTA Medical Consumption (iMCQ) and Productivity Costs (iPCQ)	3 and 6 months	Over entire 6 months follow-up: Costs (€2017): intervention=1935 (224); control=2098 (191) QALYs: intervention=0.4452 (0.0052); control=0.4435 (0.0045) Incremental costs (€): -163 (95%CI -665 to 326) Incremental QALYs: 0.0017 (-0.0121 to 0.0155); Probability that Oncokompas is more effective (60%), less costly (73%), and both more effective and less costly (47%) than usual care.
Watson 2018, **Burns 2017**	Cost-utility, Health-service resource use, Out-of-pocket expenses, Losses from inability to undertake usual activities, Costs in £	EQ-5D for effects; Patient diary to record health service contacts, over the counter meds; Days missed from usual activities	EQ-5D collected at baseline and 7 months; diary completed for 0-3, 3-6 and 6-7 months	Cost: intervention, £421.48, control, £177.75; Effect intervention: -0.0230, control -0.0335; ICER £22,950 (95%CI -12.433 to £4.850); GBP 2014 Intervention group reported less practice nurse, hospital doctor, and hospital nurse visits than control group.

[a]Though this review included 33 interventions, this table only reports those with a health economic evaluation.
[b]These health economic outcomes were for all chronic disease patients, not only cancer survivors.
EQ-5D = EuroQol-5 dimension; ICER = Incremental cost-effectiveness ratio; QALYs = Quality-adjusted life years

Supplementary Data S1

See 'Supplementary data' spreadsheet

See 'QoL findings' tab for data on quality of life

See 'Self-efficacy findings' tab for data on self-efficacy

See 'PRISMS' tab for intervention component judgement reasons
